# Cardioprotective effect of diabetic medication on cancer patients undergoing proven cardiotoxic chemotherapy: a systematic review and meta-analysis

**DOI:** 10.1186/s40959-025-00424-4

**Published:** 2026-01-15

**Authors:** Abdelrahman El-Helbawy, Sara T. Zaki, Fouad Hanna, Mohamed Bassiouni, Khaled O. Yaseen, Amany H. Ali, Oliver M. Farid

**Affiliations:** 1 Faculty of Medicine, Helwan University, Cairo, Egypt; 2 Faculty of Pharmacy, Badr University, Cairo, Egypt; 3 Faculty of Medicine, Cairo University, Giza, 11511 Egypt; 4 Faculty of Pharmacy, Mansoura University, Al Mansurah, Egypt; 5 Al-Azhar University Faculty of Medicine for Girls, Cairo, Egypt

**Keywords:** Heart failure, Cancer patients, Cardioprotective therapy, GLP-1 receptor agonists, Metformin, Diabetic medications

## Abstract

**Background:**

Multiple chemotherapeutic agents, such as anthracyclines, have recently shown a fatal potential for cardiotoxic effects in their patients. However, their efficacy is often hindered by their harmful adverse effects, mostly cardiotoxicity. DM medications such as metformin, SGLT-2 inhibitors, and GLP-1 receptor agonists have exhibited promising cardioprotective properties in rat clinical trials.

**Purpose:**

We conducted our study to investigate the observational association between diabetic medications and Cardioprotective effect in cancer patients treated with cardiotoxic chemotherapeutics.

**Methods:**

A meta-analysis following PRISMA guidelines included observational studies comparing diabetic cancer patients treated with chemotherapy. Outcomes evaluated were heart failure (HF) incidence, HF exacerbation, atrial fibrillation (AF), hospitalization, and mortality. Data were independently extracted by five investigators from multiple databases up to January 20, 2025.

**Results:**

Eight studies involving 27,015 patients were included. DM medication use may be associated with lower mortality (OR 0.50, *p* < 0.00001), HF incidence (OR 0.36, *p* = 0.007), HF exacerbation (OR 0.58, *p* < 0.0001), although heterogeneity was significant. Subgroup analysis revealed that SGLT-2 inhibitors may be associated with reduced mortality by 50%, HF incidence by 64%, and HF exacerbation by 49%. Hazard ratio analysis indicated SGLT-2 inhibitors use was associated with lower mortality risk in patients receiving cardiotoxic chemotherapy (HR 0.58, *p* = 0.001).

**Conclusion:**

Diabetic medications, particularly SGLT-2 inhibitors, may be associated with observational cardioprotective effect in cancer patients undergoing cardiotoxic chemotherapy. These findings highlight the need for further prospective trials to confirm subgroup benefits.

**Supplementary Information:**

The online version contains supplementary material available at 10.1186/s40959-025-00424-4.

## Introduction

Cardiotoxicity remains a major challenge in cardio-oncology, as chemotherapy can lead to severe cardiovascular complications. Approximately 10% of cancer patients experience fatal cardiovascular events related to treatment. The cardiotoxic effects of anticancer therapies manifest at both cellular and tissue levels [1, 2].

Anthracyclines (ANTs), including epirubicin, daunorubicin, and idarubicin, are among the most effective chemotherapeutic agents for various hematological and solid malignancies, such as breast cancer, leukemia, and lymphomas [3]. However, their clinical efficacy is frequently limited by anthracycline-induced cardiotoxicity (AIC), which can present as electrocardiographic changes, arrhythmias, myocarditis, pericarditis, myocardial infarction, cardiomyopathy, and heart failure, including congestive heart failure [4]. The primary mechanism of AIC is thought to involve inhibition of topoisomerase 2β, triggering cell death pathways and impairing mitochondrial biogenesis [5].

Recent research indicates that diabetic medications, including metformin, sodium-glucose cotransporter-2 (SGLT2) inhibitors, and glucagon-like peptide-1 (GLP-1) receptor agonists, may offer cardioprotective effects. These drugs exert pleiotropic actions—antioxidant, anti-inflammatory, and mitochondrial-protective—which may mitigate anthracycline-induced cardiotoxicity [6–8].

While preliminary evidence is promising, the collective cardioprotective effect of these antidiabetic agents in the oncology setting remains unclear. To address this gap, we conducted a systematic review and meta-analysis to determine whether diabetic medications can decrease chemotherapy-induced cardiotoxicity and improve cardiovascular outcomes in this vulnerable patient population.

## Methods

### Guidelines and ethics

This meta-analysis and systematic review was conducted in compliance with the Cochrane Handbook of Systematic Reviews of Interventions [9] and reported according to the Preferred Reporting Items of Systematic Reviews and Meta-Analyses (PRISMA 2020) guidelines [10]. The protocol ID on PROSPERO is (CRD42024612123) [11]. (Supplementary Files: PROSPERO record and PRISMA checklist)

### Study selection

An extensive literature search was conducted across five databases: PubMed, Web of Science, Scopus, ClinicalTrials.gov, and Cochrane Library, yielding a total of 395 studies. The search strategy combined keywords and Medical Subject Headings (MeSH) related to SGLT2 inhibitors, metformin, GLP-1 receptor agonists, anthracyclines, cardiotoxicity, and cancer treatment. Manual screening of reference lists from eligible studies was also performed to ensure comprehensive retrieval of relevant literature. The MeSH-based search strategy included the following terms: (anthracycline OR daunorubicin OR doxorubicin OR epirubicin OR DOX OR idarubicin) AND ((SGLT2 inhibitor OR SGLT2i OR gliflozin OR sodium-glucose transport protein OR sodium-glucose cotransporter-2 OR Jardiance OR Farxiga OR Invokana OR Brenzavvy) OR (metformin OR Glucophage OR metformin HCL OR Axpinet OR Diagemet OR Glucient OR Meta Bet)) AND (cardiotoxicity OR cardioprotective OR cardioprotect). This comprehensive search strategy was designed to minimize the risk of omitting relevant studies and to ensure inclusivity in the review process. The systematic review and meta-analysis were conducted and reported in accordance with Preferred Reporting Items for Systematic Reviews and Meta-Analyses (PRISMA) guidelines, including only studies published in English.

### Design and study selection

Following the search, a Screening process was conducted to select studies that met predefined inclusion and exclusion criteria, Endnote software was used to remove duplicate records, and the final number of records was uploaded to Rayyan for title and abstract screening [12]. All studies were screened independently by two reviewers. Full text of the potentially eligible studies were assessed using the same methodology. A third reviewer contributed to the decisions regarding questionable studies. Screening was done based on the following PICO framework:

- Population: Adult patients (≥ 18 years) with a cancer diagnosis, scheduled to receive cardiotoxic chemotherapy regimens that include anthracyclines, whether diabetic or non-diabetic.
- Intervention: Concomitant use of oral cardioprotective diabetic medications (SGLT2 inhibitors or metformin or GLP-1 receptor agonists), initiated before or during anthracycline chemotherapy.
- Comparator : Patients from the same population receiving standard anthracycline chemotherapy without concomitant use of the intervention diabetic drug (SGLT2 inhibitors, GLP-1 receptor agonists, or metformin). If the population group is diabetic, they would be controlled on other diabetic medication rather than the specific intervention drug used in the study.
- Outcomes: new onset of heart failure, HF hospitalization, AMI, ischemic stroke, arrhythmia incidence, and death.
- Study design: both observational studies and randomized controlled trials.

Excluded were animal studies, reviews, case reports, and case series, as these were not original studies.

This approach aligns with best practices in systematic reviews and meta-analyses, ensuring that our findings are based on the most applicable and reliable data available.

### Outcomes definition

Heart failure incidence in the context of cancer therapy-related cardiac dysfunction (CTRCD), by definition, according to ESC cardio-oncology (2022 guidelines), is considered as new or worsening myocardial dysfunction, often classified by left ventricular ejection fraction (LVEF) changes. Typically, HF incidence relates to a reduction in LVEF below 50% or significant deterioration in cardiac biomarkers or myocardial strain, even when asymptomatic. Concerning heart failure exacerbation, according to ESC cardio-oncology (2022 guidelines), it is a sudden worsening or acute decompensation of heart failure symptoms requiring urgent medical attention. Also, arrhythmia incidence is defined as new onset or worsening arrhythmias during or after cancer therapy with certain cancer drugs. In the table attached is a summary of the three main outcomes in each included study and a comparison to the predefined outcome according to ESC cardio-oncology guidelines [13]. (Supplementary Table: included studies outcomes definition).

### Data extraction

Two reviewers independently extracted data, including author name, year of publication, sample size, patient characteristics, study design, and outcome measures. Any discrepancies were resolved through discussion or, when necessary, consultation with a third reviewer to achieve consensus. Eligible studies were distributed among co-authors for data extraction, which was conducted using a standardized Excel spreadsheet. Extracted data included the article identification (ID), title, authors, year of publication, journal, country, study design, recruitment period, sample size, and group allocation—specifically: Diabetic cancer patients treated with anthracyclines and diabetic medications, and Diabetic cancer patients treated with anthracyclines without diabetic medications. Patient characteristics encompassed age, sex, comorbidities, and key clinical outcomes, such as heart failure (HF) incidence, hospitalization, HF exacerbation, atrial fibrillation (AF), and mortality. The data were summarized and validated by an independent co-author to ensure accuracy and completeness. Quality assessment of each included study was also performed independently by two reviewers, with disagreements resolved through team consensus or adjudication by a third reviewer.

### Strategy for data synthesis

The primary objective of our analysis was to investigate the impact of diabetic medications, specifically SGLT2 inhibitors, metformin, and GLP-1 receptor agonists, in preventing chemotherapy-induced cardiotoxicity among diabetic cancer patients treated with anthracycline-based chemotherapy, compared to diabetic cancer patients receiving similar regimens without these medications. A secondary objective was to assess and discuss the heterogeneity among studies. For dichotomous outcome variables, we calculated the odds ratio (OR) and 95% confidence intervals (CI) to compare outcomes to quantify the cardioprotective effect of the diabetic medications (SGLT2 inhibitors, metformin, and GLP-1 receptor agonists) on outcome variables. Also, we calculated the hazard ratio (HR) and 95% confidence intervals (CI) to quantify the cardioprotective effect of the diabetic medication (SGLT-2 inhibitors only) on cancer therapy-related cardiac dysfunction. A p-value less than 0.05 was considered statistically significant. Subgroup analyses were performed using two effect sizes (OR and HR) according to the class of diabetic medications (SGLT-2 inhibitors, metformin, and GLP-1 receptor agonists) in order to separate the DM medications from each other and according to cancer therapy (anthracycline vs. all cancer therapy) for assessed outcomes. We performed sensitivity analyses to evaluate the robustness of our findings. First, we excluded studies with a high risk of bias for OR outcomes and studies reporting unadjusted HRs. Second, to investigate the substantial statistical heterogeneity observed in some analyses, we conducted a leave-one-out analysis by sequentially excluding each study and recalculating the I² statistic. The consistency of the pooled effect sizes throughout these procedures would confirm the reliability of our conclusions. The 95% prediction interval was calculated for primary outcomes to explain the clinical dispersion of the outcomes. To account for potential differences in participant characteristics and research designs, we employed a random-effects model during our meta-analysis, the DerSimonian and Laird method for calculating the between-study variance (often denoted as “τ²” or “Tau²”). This approach ensures that our findings can be generalizable across various national contexts. Heterogeneity among the studies was assessed using the Q statistic, the I² test, and the associated P-value. Heterogeneity was deemed significant if the P-value was below 0.1. Also, we used prediction intervals to know how much the cardioprotective effect of SGLT-2 varies, as we did not focus on treatment effect as much as we focused on how much the drug effect varies in the true population. Our original plan included assessing publication bias if the number of included trials exceeded 10; however, due to the incomplete number of studies, we were unable to perform this assessment. We used the Review Manager 5.3 software for data synthesis. The extracted data were entered into the Review Manager by the first independent author and checked by a second independent author.

## Results

### Selection and characteristics (search results)

**Fig. 1:**
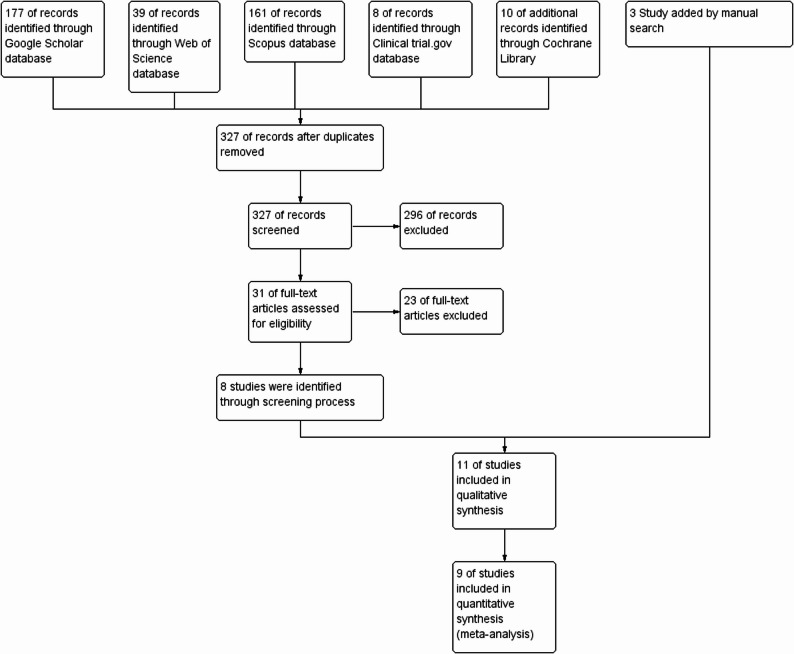
A PRISMA flow chart of our search through different databases

The search yielded a total of 395 studies: Google Scholar (*n* = 177), Web of Science (*n* = 39), Cochrane (*n* = 10), Scopus (*n* = 161), clinicaltrials.gov (*n* = 8), and, in addition, three studies identified through a manual search. All database results were imported into the Rayyan software for deduplication. After removing duplicates, 327 unique records remained for title and abstract screening. After removing duplicates (*n* = 327), 327 studies were obtained for title-and-abstract screening. Each of the two authors independently screened the studies, and any conflicts were resolved through discussion. According to our inclusion/exclusion criteria, we excluded 296 papers, and 31 studies were sought for full-text screening. The full-text screening was done, and only 8 papers were eligible for inclusion from the five databases, and three papers were obtained from a manual search. Finally, eleven studies were eligible for inclusion in Systematic review and nine studies were included in this meta-analysis after full-text review excluding studies that include non-diabetic population [7, 8, 14–22]. (Fig. 1: PRISMA flow diagram).

### Quality assessment and risk of bias

**Fig. 2:**
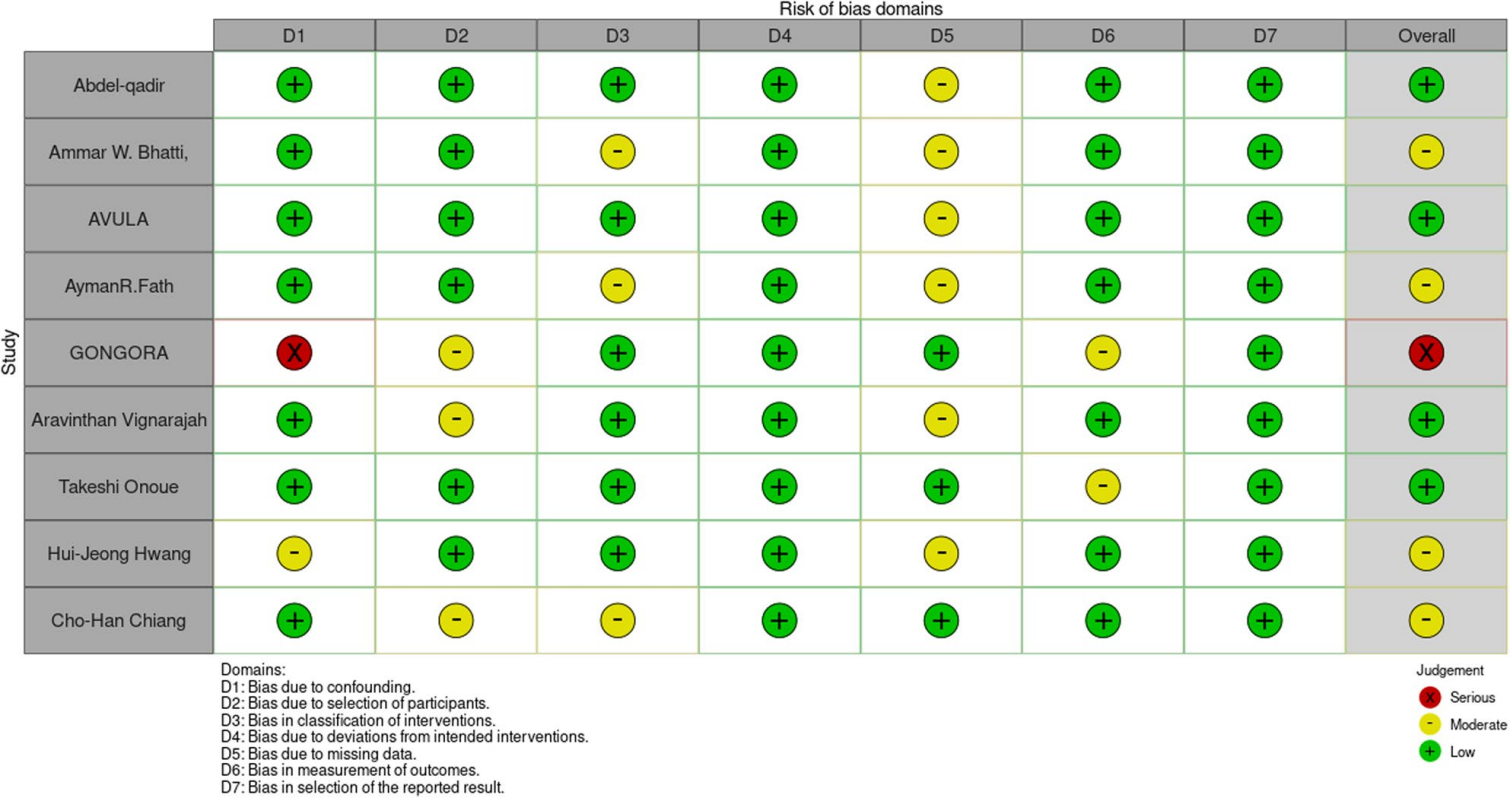
Risk of bias assessment figure

Risk of bias was assessed using the Risk of Bias in Non-randomized Studies of Interventions (ROBINS-I) tool, and the certainty of evidence was evaluated according to the Grading of Recommendations, Assessment, Development and Evaluation (GRADE) framework. Among the eight included observational studies, four were rated as low risk of bias, four as moderate risk, and one as serious risk. Overall methodological quality was acceptable; several studies demonstrated limitations related to confounding adjustment and exposure ascertainment, although others applied appropriate statistical adjustments to mitigate these issues. The study of Gongora et al. and Fath et al. exerted the greatest methodological influence on the certainty of evidence. Gongora et al. exhibited a serious risk of bias, primarily due to insufficient adjustment for baseline confounders and potential misclassification of exposure, which reduced confidence in their effect estimates. Fath et al., while rated as moderate risk, were identified as a main contributor to heterogeneity. Sensitivity analyses demonstrated that overall heterogeneity declined substantially when Fath et al. was excluded. These methodological limitations collectively led to the downgrading of GRADE certainty for several outcomes from high to moderate or low. Although several low-risk studies supported a consistent direction of effect, the limitations identified in Gongora et al. and Fath et al. highlight the importance of future prospective, well-controlled studies to confirm the cardioprotective potential of diabetic medications in patients receiving anthracycline-based chemotherapy (Fig. 2 shows ROB-1) (Table 1: GRADE assessment).

**Table 1:** GRADE assessment

Outcomes	№ of participants (studies) Follow-up	Certainty of the evidence (GRADE)	Relative effect (95% CI)	Anticipated absolute effects	
				Risk with non diabetic drug	Risk difference with Diabetic Drug
All cause of Mortality	27,015 (8 non-randomised studies)	⨁⨁⨁◯ Moderate^a, b^	OR 0.50 (0.39 to 0.63)	Study population	
				145 per 1000	67 fewer per 1000 (from 83 fewer to 48 fewer)
				Low	
				145 per 1000	67 fewer per 1000 (from 83 fewer to 48 fewer)
Atrial fibillation	22,488 (4 non-randomised studies)	⨁⨁⨁◯ Moderate^c^	OR 0.61 (0.43 to 0.87)	Study population	
				67 per 1000	25 fewer per 1000 (from 37 fewer to 8 fewer)
				Low	
				67 per 1000	25 fewer per 1000 (from 37 fewer to 8 fewer)
HF exacerbation	22,607 (5 non-randomised studies)	⨁⨁◯◯ Low^d, e^	OR 0.58 (0.45 to 0.76)	Study population	
				113 per 1000	44 fewer per 1000 (from 59 fewer to 25 fewer)
				Low	
				113 per 1000	44 fewer per 1000 (from 59 fewer to 25 fewer)
ALL cause of Hospitalization	22,488 (4 non-randomised studies)	⨁⨁◯◯ Low^f, g^	OR 0.73(0.56 to 0.94)	Study population	
				483 per 1000	78 fewer per 1000 (from 140 fewer to 15 fewer)
				Moderate	
				483 per 1000	78 fewer per 1000 (from 140 fewer to 15 fewer)
Heart Failure Incidence	20,173 (5 non-randomised studies)	⨁⨁⨁⨁ High^d, h^	OR 0.36 (0.17 to 0.76)	Study population	
				108 per 1000	66 fewer per 1000 (from 88 fewer to 24 fewer)
				Low	
				108 per 1000	66 fewer per 1000 (from 88 fewer to 24 fewer)
Incidence of Arrhythmia	3986 (3 non-randomised studies)	⨁⨁◯◯ Low^i, j^	OR 0.54 (0.23 to 1.26)	Study population	
				277 per 1000	105 fewer per 1000 (from 196 fewer to 49 more)
				Moderate	
				277 per 1000	106 fewer per 1000 (from 196 fewer to 49 more)
Myocardial Infraction	4528 (2 non-randomised studies)	⨁⨁⨁⨁ High	OR 1.49 (0.83 to 2.70)	Study population	
				7 per 1000	4 more per 1000 (from 1 fewer to 12 more)
				Low	
				7 per 1000	3 more per 1000 (from 1 fewer to 12 more)

*The risk in the intervention group (and its 95% confidence interval) is based on the assumed risk in the comparison group and the relative effect of the intervention (and its 95% CI)

*CI* Confidence interval, *OR* Odds ratio

GRADE Working Group grades of evidence

High certainty: we are very confident that the true effect lies close to that of the estimate of the effect

Moderate certainty: we are moderately confident in the effect estimate: the true effect is likely to be close to the estimate of the effect, but there is a possibility that it is substantially different

Low certainty: our confidence in the effect estimate is limited: the true effect may be substantially different from the estimate of the effect

Very low certainty: we have very little confidence in the effect estimate: the true effect is likely to be substantially different from the estimate of effect

Explanations

a. 1 study has a high risk of bias, and the other 3 studies are moderate in risk of bias

b. significant heterogeneity observed (*P* = 0.00001; I2 = 80%; τ2 = 0.08), meaning the validity of the treatment effect estimate for this pooled analysis is uncertain

c. significant heterogeneity observed (*P* < 0.0004; I2 = 83%; τ2 = 0.10). meaning the validity of the treatment effect estimate for this pooled analysis is uncertain

d. 1 study High risk of bias and other 2 studies moderate risk in bias

e. significant heterogeneity observed (*P* < 0.003; I2 = 75%; τ2 = 0.05). meaning the validity of the treatment effect estimate for this pooled analysis is uncertain

f. 2 studies are moderate in risk if bias

g. significant heterogeneity observed (*P* < 0.00001; I2 = 91%; τ2 = 0.06), meaning the validity of the treatment effect estimate for this pooled analysis is uncertain

h. significant heterogeneity observed (*P* = 0.0002; I2 = 81%; τ2 = 0.50), meaning the validity of the treatment effect estimate

i. 1 study are high risk in bias and other are moderate risk

j. significant heterogeneity observed (*P* = 0.004; I2 = 82%; τ2 = 0.38)

### Study characteristics

Ten retrospective observational studies were included in the qualitative analysis. One randomized controlled trial (RCT) and one retrospective cohort were identified and incorporated into the systematic review due to its distinct emphasis on metformin and GLP-1 receptor agonists as a cardioprotective agent in non-diabetic individuals or mixed groups of diabetic and non-diabetic patients. However, due to substantial heterogeneity in its patient population (non-diabetic), outcome measures, and comparability with the predominantly diabetic cohorts of the observational studies, it was excluded from the quantitative meta-analysis. (Table 2: Study characteristics table).

**Table 2:** Demographic data of patients

Study Author (Ref)	Country	Pub. Year	Study Design	Intervention	Propensity Score Matching (PSM)	*N* (Intervention/Non)	Adjustment Method	Covariates Adjusted	Aim of the Study	Key Summary of the Results	Follow-up
SGLT-2 Inhibitor Studies											
W. Bhatti et al. (1)	USA	2024	Retrospective Cohort	SGLT-2i	Yes: Demographics, medications, malignancies, comorbidities	8675/8675	PSM 1:1 + Multivariate Cox	11 groups (~ 40 covariates): demographics, comorbidities, medications, cancer types, laboratory values, healthcare use, antineoplastic therapies	Assess if SGLT-2i use is associated with a lower incidence of CTRCD in T2DM patients with cancer exposed to cardiotoxic agents, without prior cardiomyopathy/HF.	SGLT-2i group had a lower risk of developing CTRCD, reduced heart failure exacerbations, all-cause mortality, and all-cause hospitalizations/ED visits.	1 yr
Gongora et al. (2)	USA	2022	Retrospective Cohort	SGLT-2i	Yes: Demographics, medications, malignancies, comorbidities, baseline laboratory/echocardiographic parameters	32/96	PSM (matched 3:1)	Age, sex, anthracycline start date	Test the cardiac efficacy and overall safety of SGLT-2i in patients treated with anthracyclines.	Lower cardiac event incidence (3% vs. 20%) and lower overall mortality (9% vs. 43%) in the SGLT-2i group.	1.5 yr
Avula et al. (3)	USA	2024	Retrospective Cohort	SGLT-2i	Yes: Demographics, medications, malignancies, comorbidities, prior year healthcare use, antineoplastic therapy, radiotherapy	640/640	PSM 1:1 + Multivariate Cox	11 groups (~ 40 covariates): demographics, comorbidities, medications, cancer types, antineoplastic therapies, laboratory values, healthcare use	Examine the efficacy of SGLT-2i in patients with cancer therapy–related cardiac dysfunction (CTRCD) or HF.	SGLT-2i plus conventional therapy was associated with a lower risk of acute HF exacerbation and all-cause mortality. Less frequent all-cause hospitalizations/ED visits, AF/flutter, AKI, and need for RRT.	2 yr
Hwang et al. (4)	South Korea	2023	Retrospective Cohort	SGLT-2i	Yes: Demographics, medications, malignancies, comorbidities	779/2337	PSM 1:3 + Multivariate Cox	~ 10 covariates: demographics, comorbidities, medications, cancer types, diabetes-specific variables	Evaluate whether SGLT2i improve clinical outcomes of T2DM patients undergoing AC-containing chemotherapy.	SGLT-2i may contribute to improving clinical outcomes in patients with T2DM undergoing AC-containing chemotherapy.	3.4 yr
R. Fath et al. (5)	USA	2024	Retrospective Cohort	SGLT-2i	Yes: Demographics, medications, malignancies, comorbidities	706/706	PSM 1:1 + Multivariate Cox	~ 15 covariates: demographics, comorbidities, cardiac parameters, medications, cancer types, anthracyclines	Evaluate the safety and potential of SGLT2i for preventing cardiotoxicity in cancer patients without preexisting HF receiving anthracyclines.	Patients on SGLT-2i had lower rates of new-onset HF (HR 0.147) and arrhythmia (HR 0.397).	2 yr
Abdel-Qadir et al. (6)	Canada	2023	Retrospective Cohort	SGLT-2i	Yes: Demographics, medications, malignancies, comorbidities	99/834	ATT-PSM + Univariate Cox	~ 15 covariates: demographics, socioeconomic, diabetes-related, comorbidities, risk scoring, medications	Determine the association between SGLT-2i and cardiovascular disease (CVD) after anthracycline-containing chemotherapy.	SGLT-2i may reduce the risk of HF in patients receiving anthracycline-containing chemotherapy.	1.6 yr
Chiang et al. (7)	Taiwan	2023	Retrospective Cohort	SGLT-2i	Yes: Demographics, medications, malignancies, comorbidities	878/878	PSM 1:1 + Multivariate Cox	7 groups (~ 25 covariates): demographics, comorbidities, medications, cancer types, laboratory values, healthcare use, antineoplastic therapies	Primary outcomes: hospitalization for incident HF and all-cause mortality. Secondary outcomes: serious adverse events.	SGLT-2i recipients had a 3-fold lower rate of hospitalization for incident HF and were associated with higher overall survival (85.3% vs. 63.0% at 2 years).	2 yr
GLP-1 Receptor Agonist Studies											
Vignarajah et al. (8)	USA	2025	Retrospective Cohort	GLP-1RA	Yes: Demographics, medications, malignancies, comorbidities, prior year healthcare use, antineoplastic therapy, radiotherapy, laboratory parameters	Not available	Not available	Not available	Evaluate the impact of GLP-1 RAs on all-cause mortality, hospitalization, and heart failure exacerbation in patients with CTRCD.	Patients on GLP-1 RAs plus GDMT had a significantly lower risk of acute heart failure exacerbations, all-cause mortality, and all-cause hospitalization.	Not specified
Scalia et al. (9)	USA	2025	Retrospective Cohort	GLP-1RA	Yes: Demographics, medications, comorbidities, prior year healthcare use, laboratory parameters	201/201 (after matching)	Not available	Not available	Evaluate the role of glucagon-like peptide-1 receptor agonists (GLP-1RAs) in patients with CTRCD.	GLP-1RAs treatment was associated with a significantly lower risk of all-cause hospitalization, acute heart failure events, and acute renal failure. No difference in all-cause mortality, AF, or cardiac arrest.	Mean 295.4 days
Metformin											
Osataphan et al. (10)	Thailand	2023	Randomized, Double-Blind, Double-Dummy, Placebo-Controlled Trial	Metformin	No	Not available	No (RCT)	No (RCT)	Assess the cardioprotective effects of metformin and donepezil against doxorubicin-induced cardiotoxicity.	Neither metformin nor donepezil prevented myocardial injury. Metformin preserved mitochondrial function but lacked clinical benefit.	Not specified
Onoue et al. (11)	USA	2023	Retrospective Cohort	Metformin	Yes: Demographics, medications, malignancies, comorbidities	Not available	Not available	Not available	Test the association of metformin with the occurrence of symptomatic HF in patients with DM receiving anthracyclines.	The incidence of HF was lower in patients treated with metformin. Metformin use was also associated with lower mortality.	1 year after anthracyclines

Bibliography

1. Bhatti AW, Patel R, Dani SS, Khadke S, Makwana B, Lessey C, et al. SGLT2i and Primary Prevention of Cancer Therapy-Related Cardiac Dysfunction in Patients With Diabetes. JACC CardioOncol. 2024 Dec;6(6):863–75.

2. Gongora CA, Drobni ZD, Quinaglia Araujo Costa Silva T, Zafar A, Gong J, Zlotoff DA, et al. Sodium-Glucose Co-Transporter-2 Inhibitors and Cardiac Outcomes Among Patients Treated With Anthracyclines. JACC Heart Fail. 2022 Aug;10(8):559–67.

3. Avula V, Sharma G, Kosiborod MN, Vaduganathan M, Neilan TG, Lopez T, et al. SGLT2 Inhibitor Use and Risk of Clinical Events in Patients With Cancer Therapy-Related Cardiac Dysfunction. JACC Heart Fail. 2024 Jan;12(1):67–78.

4. Hwang H-J, Kim M, Jun JE, Yon DK. Sodium-glucose cotransporter-2 inhibitors improve clinical outcomes in patients with type 2 diabetes mellitus undergoing anthracycline-containing chemotherapy: an emulated target trial using nationwide cohort data in South Korea. Sci Rep. 2023 Dec 8;13(1):21,756.

5. Fath AR, Aglan M, Aglan A, Chilton RJ, Trakhtenbroit A, Al-Shammary OA, et al. Cardioprotective Potential of Sodium-Glucose Cotransporter-2 Inhibitors in Patients With Cancer Treated With Anthracyclines: An Observational Study. Am J Cardiol. 2024 Jul 1;222:175–82.

6. Abdel-Qadir H, Carrasco R, Austin PC, Chen Y, Zhou L, Fang J, et al. The Association of Sodium-Glucose Cotransporter 2 Inhibitors With Cardiovascular Outcomes in Anthracycline-Treated Patients With Cancer. JACC CardioOncol. 2023 Jun;5(3):318–28.

7. Chiang C-H, Chiang C-H, Chiang C-H, Ma KS-K, Peng C-Y, Hsia YP, et al. Impact of sodium-glucose cotransporter-2 inhibitors on heart failure and mortality in patients with cancer. Heart. 2023 Feb 23;109(6):470–7.

8. Vignarajah A, Kim S, Albliwi M, Ahn HM, Izda A, Naffa F, et al. The Role of GLP-1 Receptor Agonists in Managing Cancer Therapy-Related Cardiac Dysfunction. medRxiv. 2025 Jan 3;

9. Scalia IG, Ibrahim R, Abdelnabi M, Pham HN, Farina JM, Pietri MP, et al. Glucagon-like peptide-1 receptor agonists in patients with anthracycline related cardiac dysfunction. Cardiooncology. 2025 Sep 25;11(1):83.

10. Osataphan N, Phrommintikul A, Leemasawat K, Somwangprasert A, Apaijai N, Suksai S, et al. Effects of metformin and donepezil on the prevention of doxorubicin-induced cardiotoxicity in breast cancer: a randomized controlled trial. Sci Rep. 2023 Aug 7;13(1):12,759.

11. Onoue T, Kang Y, Lefebvre B, Smith AM, Denduluri S, Carver J, et al. The association of metformin with heart failure in patients with diabetes mellitus receiving anthracycline chemotherapy. JACC CardioOncol. 2023 Oct;5(5):674–82.

### Baseline characteristics

A total of 29,014 patients were included across all eleven studies, comprising 13,493 patients in the diabetic medication group and 15,521 patients in the control group. A total of 12,938 patients (44.6%) were female. Hypertension was the most common comorbidity, reported in 20,060 patients (69.1%) across ten studies. The included populations encompassed patients with various types of cancer, most frequently lymphoma, breast cancer, and genitourinary malignancies. Nearly all patients were treated with anthracyclines, with one study also including a range of other chemotherapeutic agents. Concurrent cardiovascular medications reported across studies included angiotensin receptor blockers (ARBs), angiotensin-converting enzyme (ACE) inhibitors, beta-blockers, statins, antithrombotic agents, aspirin, calcium channel blockers, and diuretics. (Table 3: Baseline Characteristics).

**Table 3:** Study characteristics table

Study	Patient (*N*)	Female (*N*)	Hypertension (*N*)	Anti-Diabetic Medication	Group with Cardioprotective Diabetic Medications (*N*)	Control Group (*N*)	Cancer Types	Chemotherapy Family	Cardiovascular Medications
SGLT-2 Inhibitors									
W. Bhatti et al. (1)	17,350	7250	12,571	SGLT-2 inhibitors	8675	8675	lymphoma-breast-genitourinary-GIT Myelodysplastic syndromes: - Mesothelial and soft tissue - Respiratory and intrathoracic - Gynecological-malignant neoplasms of lymphoid, hematopoietic, and related tissue	Anthracyclines, Antimetabolites, Monoclonal antibodies, Small-molecule TKIs, Proteasome Inhibitors, Alkylating agents, and Aromatase inhibitors	(Angiotensin antagonist ARBS)-(ACE inhibitors)-(Beta-blockers)-(statin)-(RAAS inhibitors)
Gongora et al. (2)	128	57	79	SGLT-2 inhibitors	32	96	lymphoma - breast - genitourinary - GIT- Sarcoma - leukemia	Anthracyclines	(Angiotensin antagonist ARBS)- (Beta-blockers)-(statin)-(Aspirin)-(dyslipidemia medications)
Avula et al. (3)	1280	532	1215	SGLT-2 inhibitors	640	640	lymphoma-breast-genitourinary-GIT-myelodysplastic syndromes -Mesothelial and soft tissue -Respiratory and intrathoracic -Gynecological -Malignant neoplasms of lymphoid, hematopoietic, and related tissue	Alkylating agents, Anthracenediones, Anthracyclines, Antimetabolites, Aromatase inhibitors, Monoclonal antibodies, Proteosome inhibitors, Small-molecule TKIs, and Radiation therapy.	(Antiarrhythmics)-(Antilipemic agents)
Hwang et al. (4)	3116	2260	1453	SGLT-2 inhibitors	779	2337	lymphoma—breast—genitourinary	Anthracyclines, Alkylating agents, Antimicrotubule agents	(Beta-blockers)-(statin)-(Antithrombotic agents)
R. Fath et al. (5)	1412	742	1093	SGLT-2 inhibitors	706	706	lymphoma—breast—genitourinary—male genital organs—GIT—skin—mesothelial and soft tissue—respiratory and intrathoracic organs -Endocrine glands -neuroendocrine -bone and cartilage -CNS -gynecological -oropharynx	Anthracyclines	(Angiotensin antagonist ARBs) -(ACE inhibitors)-(Beta-blockers)-(statin)-(Antithrombotic agents)-(Aspirin)-(Ca-channel blocker)-(diuretics: thiazides and loop diuretics)
Abdel-Qadir et al. (6)	933	612	-	SGLT-2 inhibitors	99	834	-	Anthracyclines	-
Chiang et al. (7)	1756	825	1319	SGLT-2 inhibitors	878	878	Gastrointestinal - Genitourinary -Thoracic -Head and neck -Breast -Hematologic -Skin -Metastatic disease	Alkylating agents, Antimetabolites, Platinum, Plant alkaloids, Anthracyclines, Tyrosine kinase inhibitors, HER2 inhibitors, VEGF inhibitors, Immune checkpoint inhibitors	metformin, insulin, ACEI/ARB, Beta-blockers, Diuretics, Calcium channel blockers, Statin, Aspirin
GLP-1 Receptor Agonists									
Vignarajah et al. (8)	2446	1162	2377	GLP-1 receptor agonists	1223	1223	breast, myelodysplastic syndromes, genitourinary—male genital organs -GIT-Respiratory and intrathoracic organs -Endocrine glands-malignant neoplasms of lymphoid, hematopoietic, and related tissue	Anthracyclines, alkylating agents, antimetabolites, monoclonal antibodies, small-molecule tyrosine kinase inhibitors, and proteasome inhibitors.	(Beta-blockers)-(Antiarrhythmics)-(Antilipemic agents)-(RAAS inhibitors)
Scalia et al. (9)	402	223	387	GLP-1 agonists	201	201	N\A	Anthracyclines	(Statins)-(Antilipemic agents)- (CCB)-(Platelet Aggregation inhibitors)-(Anticoagulants)- (Antiarrhythmics)- (Digoxin)-(Metformin)-(Insulin)
Metformin									
Osataphan et al. (10)	143	143	32	Metformin	43	100	Breast	Anthracyclines	(Angiotensin antagonist ARBS)-(ACE inhibitors)-(Ca-channel blocker)
Onoue et al. (11)	350	232	455	Metformin	175	175	lymphoma-breast- Sarcoma -leukemia	Anthracyclines	(ACE inhibitors)-(Beta-blockers)-(statin)

Bibliography

1. Bhatti AW, Patel R, Dani SS, Khadke S, Makwana B, Lessey C, et al. SGLT2i and Primary Prevention of Cancer Therapy-Related Cardiac Dysfunction in Patients With Diabetes. JACC CardioOncol. 2024 Dec;6(6):863–75.

2. Gongora CA, Drobni ZD, Quinaglia Araujo Costa Silva T, Zafar A, Gong J, Zlotoff DA, et al. Sodium-Glucose Co-Transporter-2 Inhibitors and Cardiac Outcomes Among Patients Treated With Anthracyclines. JACC Heart Fail. 2022 Aug;10(8):559–67.

3. Avula V, Sharma G, Kosiborod MN, Vaduganathan M, Neilan TG, Lopez T, et al. SGLT2 Inhibitor Use and Risk of Clinical Events in Patients With Cancer Therapy-Related Cardiac Dysfunction. JACC Heart Fail. 2024 Jan;12(1):67–78.

4. Hwang H-J, Kim M, Jun JE, Yon DK. Sodium-glucose cotransporter-2 inhibitors improve clinical outcomes in patients with type 2 diabetes mellitus undergoing anthracycline-containing chemotherapy: an emulated target trial using nationwide cohort data in South Korea. Sci Rep. 2023 Dec 8;13(1):21,756.

5. Fath AR, Aglan M, Aglan A, Chilton RJ, Trakhtenbroit A, Al-Shammary OA, et al. Cardioprotective Potential of Sodium-Glucose Cotransporter-2 Inhibitors in Patients With Cancer Treated With Anthracyclines: An Observational Study. Am J Cardiol. 2024 Jul 1;222:175–82.

6. Abdel-Qadir H, Carrasco R, Austin PC, Chen Y, Zhou L, Fang J, et al. The Association of Sodium-Glucose Cotransporter 2 Inhibitors With Cardiovascular Outcomes in Anthracycline-Treated Patients With Cancer. JACC CardioOncol. 2023 Jun;5(3):318–28.

7. Chiang C-H, Chiang C-H, Chiang C-H, Ma KS-K, Peng C-Y, Hsia YP, et al. Impact of sodium-glucose cotransporter-2 inhibitors on heart failure and mortality in patients with cancer. Heart. 2023 Feb 23;109(6):470–7.

8. Vignarajah A, Kim S, Albliwi M, Ahn HM, Izda A, Naffa F, et al. The Role of GLP-1 Receptor Agonists in Managing Cancer Therapy-Related Cardiac Dysfunction. medRxiv. 2025 Jan 3;

9. Scalia IG, Ibrahim R, Abdelnabi M, Pham HN, Farina JM, Pietri MP, et al. Glucagon-like peptide-1 receptor agonists in patients with anthracycline related cardiac dysfunction. Cardiooncology. 2025 Sep 25;11(1):83.

10. Osataphan N, Phrommintikul A, Leemasawat K, Somwangprasert A, Apaijai N, Suksai S, et al. Effects of metformin and donepezil on the prevention of doxorubicin-induced cardiotoxicity in breast cancer: a randomized controlled trial. Sci Rep. 2023 Aug 7;13(1):12,759.

11. Onoue T, Kang Y, Lefebvre B, Smith AM, Denduluri S, Carver J, et al. The association of metformin with heart failure in patients with diabetes mellitus receiving anthracycline chemotherapy. JACC CardioOncol. 2023 Oct;5(5):674–82.

### Outcomes of DM medications

For outcomes expressed as odds ratios (ORs), subgroup analyses were conducted to evaluate whether the class of diabetic medication (SGLT2 inhibitors, metformin, or GLP-1 receptor agonists) influenced the cardioprotective effect compared with control groups across the assessed outcomes.

### All cause mortality

**Fig. 3:**
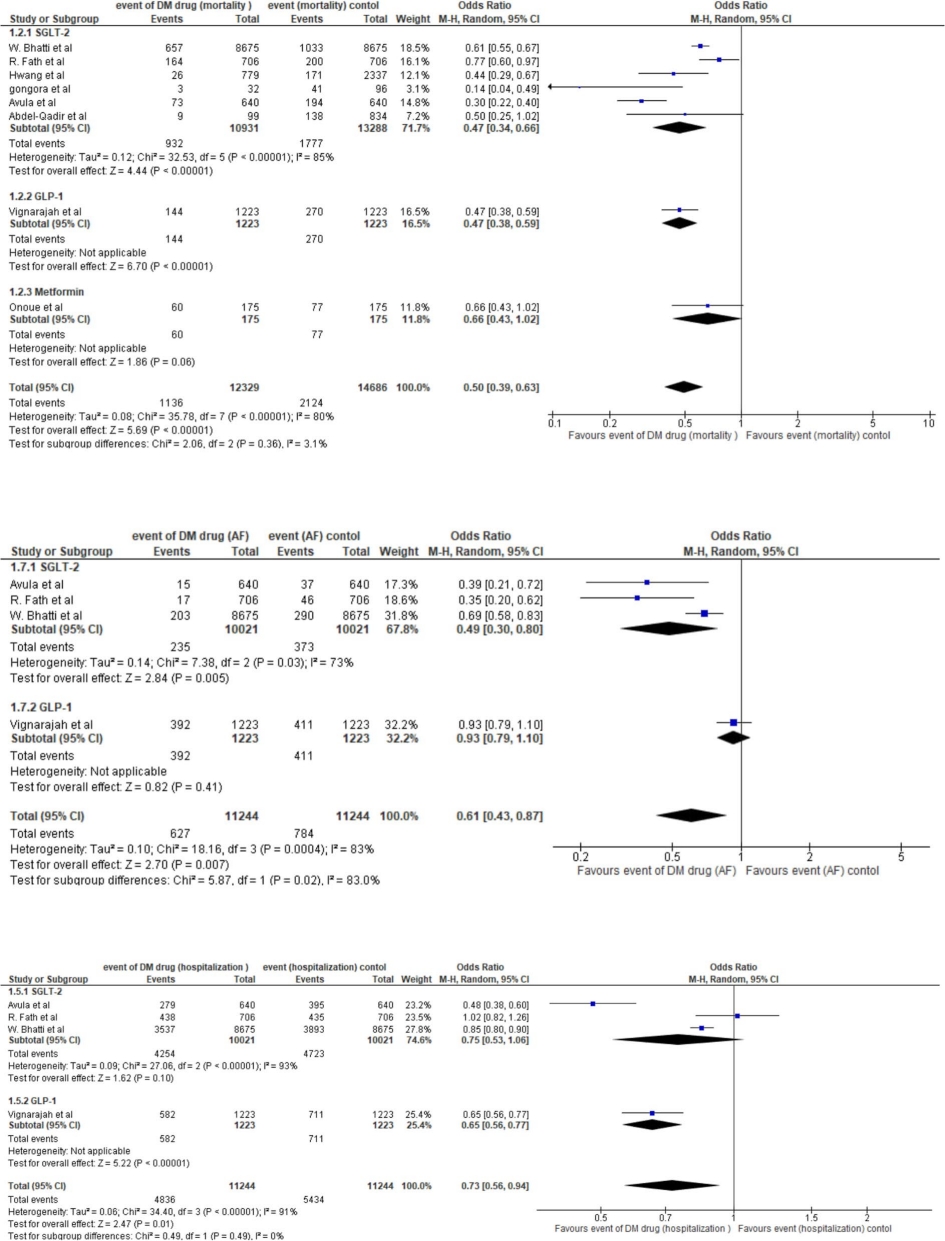
Forest plot for OR of the following all-cause mortality, incidence of atrial fibrillation and all-cause hospitalization

We included eight studies (27,015 patients) that compared the all-cause mortality risk between DM drugs (SGLT2 inhibitors or metformin or GLP-1 receptor agonist) and control (non-SGLT2 inhibitors or non-metformin or non-GLP-1 receptor agonists) groups. Each subgroup demonstrated a potential advantage of DM drugs with a reduced risk of all-cause mortality. A far smaller number of trials and participants contributed data to the metformin and GLP-1 receptor agonists subgroups (one trial for each one, 2446 participants for the GLP-1 receptor agonists subgroup, and 350 participants for the metformin subgroup) than to the SGLT-2 inhibitors subgroup (6 trials, 24219 participants), meaning that the analysis is unpowered to detect meaningful difference findings. Regarding the pooled SGLT-2 inhibitors subgroup, it was associated with a statistically significant 53% reduction in the probability of all-cause mortality compared to the control subgroup with an odds ratio (OR) of 0.47, with a 95% CI of 0.34 to 0.66 (*P* < 0.00001). There was also significant heterogeneity observed among the SGLT-2 inhibitors subgroup (*P* < 0.00001; I² = 85%; τ² = 0.12). The 95% prediction interval was 0.11 to 2.07. In the pooled analysis, a total of 3260 patients died, 1136 in the DM group and 2124 in the control group. The pooled OR under the random-effects model showed that the probability of all-cause mortality was associated with a significantly lower rate in the DM drugs group compared to the control group, with an OR of 0.50, with a 95% CI of 0.39 to 0.63, p-value < 0.00001, representing a 50% (half the odds) reduction in the odds of mortality. There was also significant heterogeneity observed (*P* = 0.00001; I² = 80%; τ² = 0.08). The prediction interval in 95% of all comparable populations falls in the interval 0.24 to 1.06. Sensitivity analyses consistently supported these significant findings (OR 0.48; 95% CI, 0.37 to 0.62; *P* < 0.00001; participants = 25,475; studies = 6; I² = 79%). (Fig. 3: Forest plot A-all cause mortality).

### Incidence of atrial fibrillation

We included all four studies (22,488 patients) that compared atrial fibrillation (AF) between DM drug (SGLT-2 inhibitors or GLP-1 receptor agonists) and control (non-SGLT-2 inhibitors or non-GLP-1 receptor agonists) groups. A far smaller number of trials and participants contributed data to the GLP-1 receptor agonists subgroup (one trial, 2446 participants for the GLP-1 subgroup, 350 participants for the metformin subgroup) than to the SGLT-2 inhibitors subgroup (three trials, 20042 participants), indicating that the analysis was unpowered to detect meaningful differences. Regarding the SGLT-2 inhibitors subgroup, it was associated with a significantly lower probability of AF than the control subgroup, with an odds ratio (OR) of 0.49 (95% CI 0.30–0.80, *p* = 0.005), representing a 51% reduction in the odds of atrial fibrillation. Significant heterogeneity was also observed among the SGLT-2 inhibitors subgroups (*P* = 0.03; I² = 73%; τ² = 0.14). In the pooled analysis, 1411 patients developed AF, including 627 in the DM group and 784 in the control group. The pooled OR under the random-effects model showed that the DM group was associated with significantly lower rates of AF than the control group (OR of 0.61; 95% CI, 0.43–0.87; *p* = 0.007), representing a 39% reduction in the odds of AF. Significant heterogeneity was also observed (*P* = 0.0004; I² = 83%; τ² = 0.10). Sensitivity analyses consistently supported these significant findings (OR 0.71; 95% CI, 0.51 to 0.99; *P* = 0.04; participants = 21,076; studies = 3; I² = 81%). (Fig. 3: Forest plot B- Incidence of Atrial Fibrillation).

### All-cause hospitalization

​ We included four studies (22488 patients) that compared all-cause hospitalization between DM drug (SGLT-2 inhibitors or GLP-1 receptor agonists) and control (non-SGLT-2 inhibitor or non-GLP-1) groups. Each subgroup study demonstrated the potential advantages of DM drugs with a reduced risk of all-cause hospitalization. A far smaller number of trials and participants contributed data to the GLP-1 receptor agonists subgroups (one trial, 2446 participants for the GLP-1 receptor agonists subgroup) than to the SGLT-2 inhibitors subgroup (three trials, 20042 participants), indicating that the analysis was unpowered to detect meaningful differences. Regarding the SGLT-2 inhibitors subgroup, it was associated with 25% lower rates of all-cause hospitalization than the control group, although there was no statistical significance between the two groups [(OR) of 0.75; 95% CI, 0.53–1.06; *p* = 0.10]. There was also significant heterogeneity observed among the SGLT-2 inhibitor subgroup (*P* < 0.00001; I² = 93%; τ² = 0.09). In the pooled analysis, 10,270 patients developed all-cause hospitalization, 4836 in the DM group and 5434 patients in the control group. The pooled OR under the random-effects model showed that the DM group was associated with a significantly lower probability of all-cause hospitalization than the control group (OR: 0.73; 95% CI: 0.56–0.94; *p* = 0.01), representing a 27% reduction in the odds of hospitalization. Significant heterogeneity was also observed (*P* < 0.00001; I² = 91%; τ² = 0.06). Sensitivity analyses consistently supported these significant findings (OR 0.65; 95% CI, 0.48 to 0.89; *P* = 0.007; participants = 21,076; studies = 3; I² = 93%). (Fig. 3: Forest plot C- All-Cause Hospitalization).

### Heart failure exacerbation

**Fig. 4:**
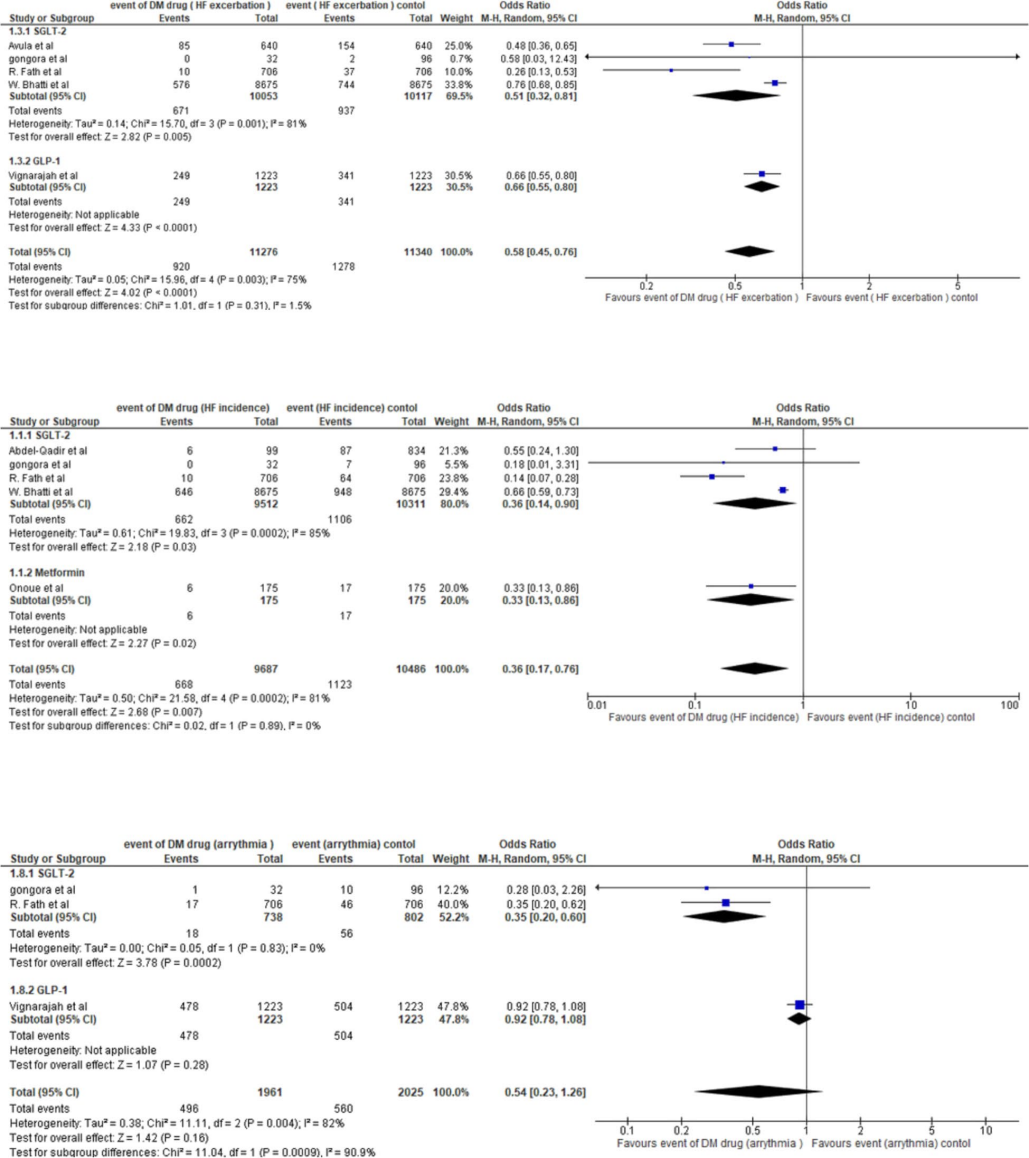
Forest plot 2 for OR of the following heart failure exacerbation, heart failure incidence and incidence of arrhythmia

We included five studies (22,616 patients) that compared heart failure (HF) exacerbation between DM drug (SGLT2 inhibitors, GLP-1 receptor agonists, or metformin) and control (non-SGLT-2 inhibitors, non-GLP-1 receptor agonists, or non-metformin) groups. A far smaller number of trials and participants contributed data to the GLP-1 receptor agonists subgroup (one trial, 2,446 participants) than to the SGLT-2 inhibitors subgroup (four trials, 20,170 participants), indicating that the analysis was unpowered to detect meaningful differences. Regarding the SGLT-2 inhibitors subgroup, it was associated with a significantly lower probability of HF exacerbation than the control subgroup, with an odds ratio (OR) of 0.51 (95% CI 0.32–0.81, *p* < 0.001), representing a 49% reduction in the odds of HF exacerbation. Significant heterogeneity was also observed among the SGLT-2 inhibitors subgroups (*P* < 0.001; I² = 91%; τ² = 0.14). In the combined analysis, 2198 patients experienced HF exacerbation, comprising 920 from the DM group and 1278 from the control group. The pooled OR under the random-effects model showed that the DM group was associated with significantly lower rates of HF exacerbation than the control group (OR 0.58; 95% CI, 0.45–0.76; *p* < 0.0001), representing a 42% reduction in the odds of HF exacerbation. Significant heterogeneity was also observed (*P* = 0.003; I² = 75%; τ² = 0.05). The prediction interval in 95% of all comparable populations falls in the interval 0.25 to 1.34. Sensitivity analyses consistently supported these significant findings (OR 0.65; 95% CI, 0.52 to 0.81; *P* = 0.0001; participants = 21,076; studies = 3; I² = 76%). (Fig. 4: Forest plot D-Heart Failure Exacerbation).

### Heart failure incidence

We included five studies (20,173 patients) that compared heart failure (HF) incidence between DM drug (SGLT-2 inhibitors or metformin) and control (non-SGLT-2 inhibitors or non-metformin) groups. A far smaller number of trials and participants contributed data to the metformin subgroup (350 participants) than to the SGLT-2 inhibitor subgroup (four trials, 19,823 participants), indicating that the analysis was unpowered to detect meaningful differences. Regarding the SGLT-2 inhibitors subgroup, it was associated with a significantly lower probability of HF incidence than the control subgroup, with an odds ratio (OR) of 0.36 (95% CI 0.14–0.90, *p* = 0.03), representing a 64% reduction in the odds of HF incidence. Significant heterogeneity was also observed among the SGLT-2 subgroups (*P* = 0.0002; I² = 85%; τ² = 0.61). In the pooled analysis, 1791 patients developed HF incidence, including 668 in the DM group and 1123 in the control group. The pooled OR under the random-effects model showed that the DM group was associated with significantly lower rates of HF incidence than the control group (OR 0.36; 95% CI, 0.17–0.76; *p* = 0.007), representing a 64% reduction in the odds of HF incidence. Significant heterogeneity was also observed (*P* = 0.0002; I² = 81%; τ² = 0.50). The prediction interval in 95% of all comparable populations falls in the interval 0.03 to 4.64. (Fig. 4: Forest plot E- Heart Failure Incidence).

### Incidence of arrhythmia

We included three studies (3,986 patients) that compared arrhythmia between the DM drug (SGLT-2 inhibitors or GLP-1 receptor agonists) and control (non-SGLT-2 inhibitors or non-GLP-1 receptor agonists) groups. A far smaller number of trials and participants contributed data to the SGLT-2 inhibitor subgroup (two trials, 1,540 participants) than to the GLP-1 receptor agonists subgroup (one trial, 2,446 participants), indicating that the analysis was unpowered to detect meaningful differences. Regarding the SGLT-2 inhibitors subgroup, it was associated with a significantly lower probability of arrhythmia than the control subgroup, with an odds ratio (OR) of 0.35 (95% CI 0.20–0.60, *p* = 0.0002), representing a 65% reduction in the odds of arrhythmia. No heterogeneity was observed among the SGLT-2 inhibitor subgroups (*P* = 0.83; I² = 0%; τ² = 0.00). In the pooled analysis, 1056 patients developed arrhythmia, including 496 in the DM group and 560 in the control group. The pooled OR under the random-effects model showed no statistically significant difference in arrhythmia rates between the DM group and the control group (OR 0.54; 95% CI, 0.23–1.26; *p* = 0.16). Significant heterogeneity was observed in the pooled analysis (*P* = 0.004; I² = 82%; τ² = 0.38). (Fig. 4: Forest plot F-Incidence of Arrhythmia).

### Hazard ratio outcomes

For effect size hazard ratio (HR), a subgroup analysis was performed to test whether being assessed to anthracycline or all cancer therapy, including anthracycline, modifies the cardioprotective effect of SGLT-2 inhibitors in comparison to non-SGLT-2 inhibitors regarding the probability of the following outcomes. (Table 4: Summary of HR outcomes).

**Table 4:** Summary of HR outcome

Outcome/Subgroup/Study	Follow-up	*N* (SGLT-2i/Non)	HR (95% CI)*	*P*-value	Adjustment	Analysis Method
ALL-CAUSE MORTALITY						
ANTHRACYCLINE-ONLY COHORT						
Gongora 2022 (1)	1.5 yr	32/96	0.21 (0.07–0.67)	0.005	Unadjusted	Univariate KM + PSM
Fath 2024 (2)	2 yr	706/706	0.90 (0.73–1.11)	0.310 (NS)	Adjusted	Multivariate Cox + PSM
Abdel-Qadir 2023 (3)	1.6 yr	99/834	0.63 (0.36–1.11)	0.11 (NS)	Adjusted	Univariate Cox + ATT-PSM
Hwang 2023 (4)	3.4 yr	779/2,337	0.42 (0.28–0.63)	0.001	Adjusted	Multivariate Cox + PSM
ALL CANCER THERAPY COHORT						
Avula 2024 (5)	2 yr	640/640	0.476 (0.363–0.623)	0.001	Adjusted	Multivariate Cox + PSM
Chiang 2023 (6)	2	878\878	0.35 (0.28 0.43)	< 0.001	Adjusted	Multivariate Cox + PSM
Bhatti 2024 (7)	1 yr	8,675/8,675	0.67 (0.61–0.74)	0.001	Adjusted	Multivariate Cox + PSM
HEART FAILURE INCIDENCE/NEW-ONSET HF						
ANTHRACYCLINE-ONLY COHORT						
Gongora 2022 (1)	1.5 yr	32/96	0.06 (0.01–0.24)	0.001	Unadjusted	Univariate KM + PSM
Fath 2024 (2)	2 yr	706/706	0.15 (0.07–0.32)	0.001	Adjusted	Multivariate Cox + PSM
Abdel-Qadir 2023 (3)	1.6 yr	99/834	0.55 (0.23–1.32)		Adjusted	Univariate Cox + ATT-PSM
ALL CANCER THERAPY COHORT						
Bhatti 2024 (7)	1 yr	8,675/8,675	0.76 (0.69–0.84)	0.001	Adjusted	Multivariate Cox + PSM
HEART FAILURE EXACERBATION						
ANTHRACYCLINE-ONLY COHORT						
Gongora 2022 (1)	1.5 yr	32/96	0.06 (0.01–0.24)	0.001	Unadjusted	Univariate KM + PSM
Fath 2024 (2)	2 yr	706/706	0.06 (0.01–0.36)	0.001	Adjusted	Multivariate Cox + PSM
ALL CANCER THERAPY COHORT						
Avula 2024 (5)	2 yr	640/640	0.618 (0.474–0.806)	0.001	Adjusted	Multivariate Cox + PSM
Bhatti 2024 (7)	1 yr	8,675/8,675	0.81 (0.72–0.90)	0.001	Adjusted	Multivariate Cox + PSM
ATRIAL FIBRILLATION						
ALL CANCER THERAPY COHORT						
Avula 2024 (5)	2 yr	640/640	0.518 (0.284–0.947)	0.030	Adjusted	Multivariate Cox + PSM
Bhatti 2024 (7)	1 yr	8,675/8,675	0.74 (0.62–0.89)	0.001	Adjusted	Multivariate Cox + PSM
HOSPITALIZATION						
ALL CANCER THERAPY COHORT						
Avula 2024 (5)	2 yr	640/640	0.720 (0.618–0.840)	0.001	Adjusted	Multivariate Cox + PSM
Bhatti 2024 (7)	1 yr	8,675/8,675	0.93 (0.89–0.93)		Adjusted	Multivariate Cox + PSM
Chiang 2023 (6)	2	878\878	0.28 (0.11 0.77)	0.013	Adjusted	Multivariate Cox + PSM
ANTHRACYCLINE-ONLY COHORT						
Fath 2024 (2)	2 yr	706/706	1.1 (0.96–1.26)		Adjusted	Multivariate Cox + PSM

Bibliography

1. Gongora CA, Drobni ZD, Quinaglia Araujo Costa Silva T, Zafar A, Gong J, Zlotoff DA, et al. Sodium-Glucose Co-Transporter-2 Inhibitors and Cardiac Outcomes Among Patients Treated With Anthracyclines. JACC Heart Fail. 2022 Aug;10(8):559–67.

2. Fath AR, Aglan M, Aglan A, Chilton RJ, Trakhtenbroit A, Al-Shammary OA, et al. Cardioprotective Potential of Sodium-Glucose Cotransporter-2 Inhibitors in Patients With Cancer Treated With Anthracyclines: An Observational Study. Am J Cardiol. 2024 Jul 1;222:175–82.

3. Abdel-Qadir H, Carrasco R, Austin PC, Chen Y, Zhou L, Fang J, et al. The Association of Sodium-Glucose Cotransporter 2 Inhibitors With Cardiovascular Outcomes in Anthracycline-Treated Patients With Cancer. JACC CardioOncol. 2023 Jun;5(3):318–28.

4. Hwang H-J, Kim M, Jun JE, Yon DK. Sodium-glucose cotransporter-2 inhibitors improve clinical outcomes in patients with type 2 diabetes mellitus undergoing anthracycline-containing chemotherapy: an emulated target trial using nationwide cohort data in South Korea. Sci Rep. 2023 Dec 8;13(1):21,756.

5. Avula V, Sharma G, Kosiborod MN, Vaduganathan M, Neilan TG, Lopez T, et al. SGLT2 Inhibitor Use and Risk of Clinical Events in Patients With Cancer Therapy-Related Cardiac Dysfunction. JACC Heart Fail. 2024 Jan;12(1):67–78.

6. Chiang C-H, Chiang C-H, Chiang C-H, Ma KS-K, Peng C-Y, Hsia YP, et al. Impact of sodium-glucose cotransporter-2 inhibitors on heart failure and mortality in patients with cancer. Heart. 2023 Feb 23;109(6):470–7.

7. Bhatti AW, Patel R, Dani SS, Khadke S, Makwana B, Lessey C, et al. SGLT2i and Primary Prevention of Cancer Therapy-Related Cardiac Dysfunction in Patients With Diabetes. JACC CardioOncol. 2024 Dec;6(6):863–75.

### Hazard ratio of mortality

**Fig. 5:**
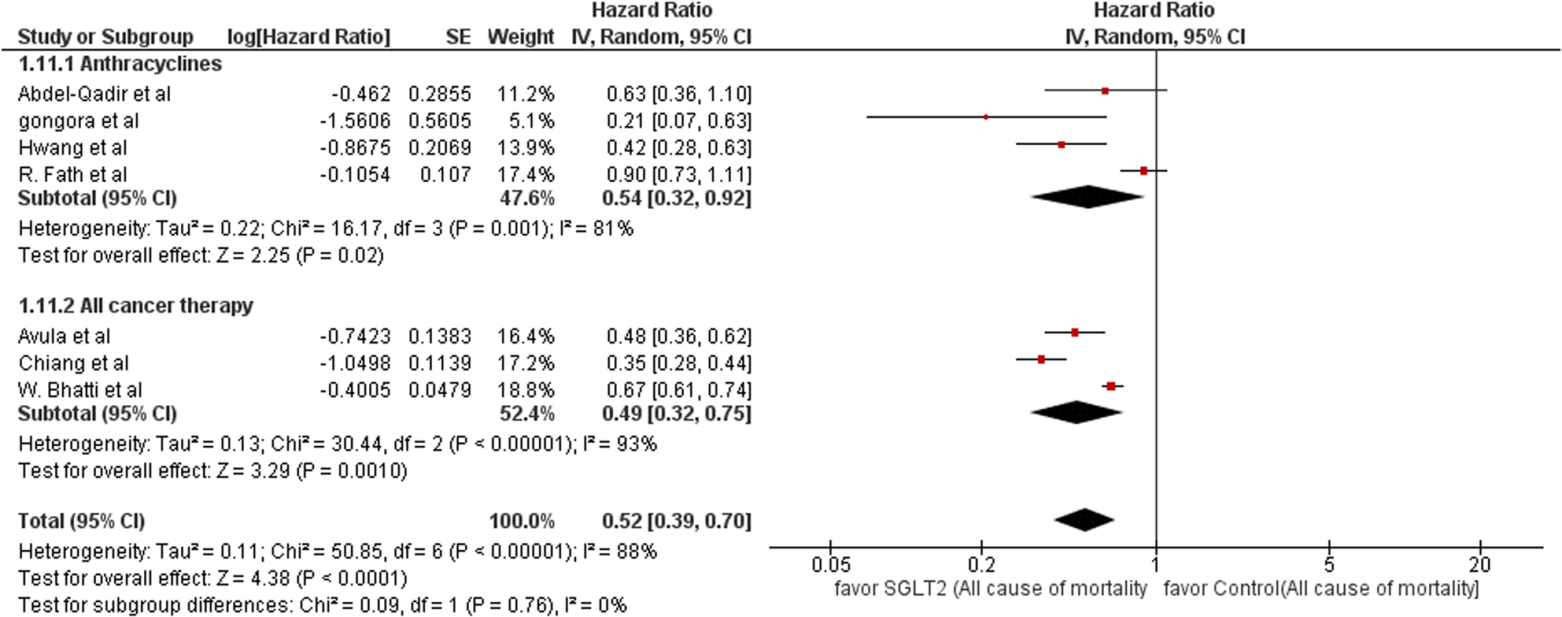
Forest plot of all cause mortality HR

We included seven studies that compared the hazard ratio of mortality between SGLT-2 inhibitors and control (non-SGLT-2 inhibitors). Each subgroup demonstrated a potential advantage of SGLT-2 inhibitors with a reduced risk of mortality, either assessed to anthracycline or all cancer therapy. The mean effect (HR) for the anthracycline subgroup was 0.54 with a CI of 0.32 to 0.92. The mean effect (HR) for the all cancer therapy subgroup was 0.49 with a CI of 0.32 to 0.75. The test for subgroup differences between groups yields (df = 1, *p* = 0.76), indicating that there is no statistically significant subgroup effect. However, a small number of trials and participants contributed data for both the anthracycline subgroup (3 trials) and all cancer therapy subgroup (3 trials), meaning that the analysis was unpowered to detect meaningful differences. In the pooled analysis, the SGLT-2 inhibitor group was associated with decreased risk of mortality compared to the non-SGLT-2 inhibitors group in patients exposed to all cancer therapy and anthracycline alone, with a hazard ratio (HR) of 0.52, 95% CI: 0.39–0.70; *P* < 0.0001. There was also significant heterogeneity observed (*P* < 0.00001; I² = 88%; τ² = 0.11). The 95% prediction interval was (0.17, 1.59). Sensitivity analyses were done, and heterogeneity remains significant (HR 0.49; 95% CI, 0.36 to 0.68; *P* < 0.0001; studies = 5; I²=88%) but is resolved in one of the two subgroups (anthracycline subgroup) (HR 0.49; 95% CI, 0.33 to 0.72; *P* = 0.0003; studies = 2; I²=24%). (Fig. 5: Forest plot of all cause mortality HR).

### Hazard ratio of HF incidence

**Fig. 6:**
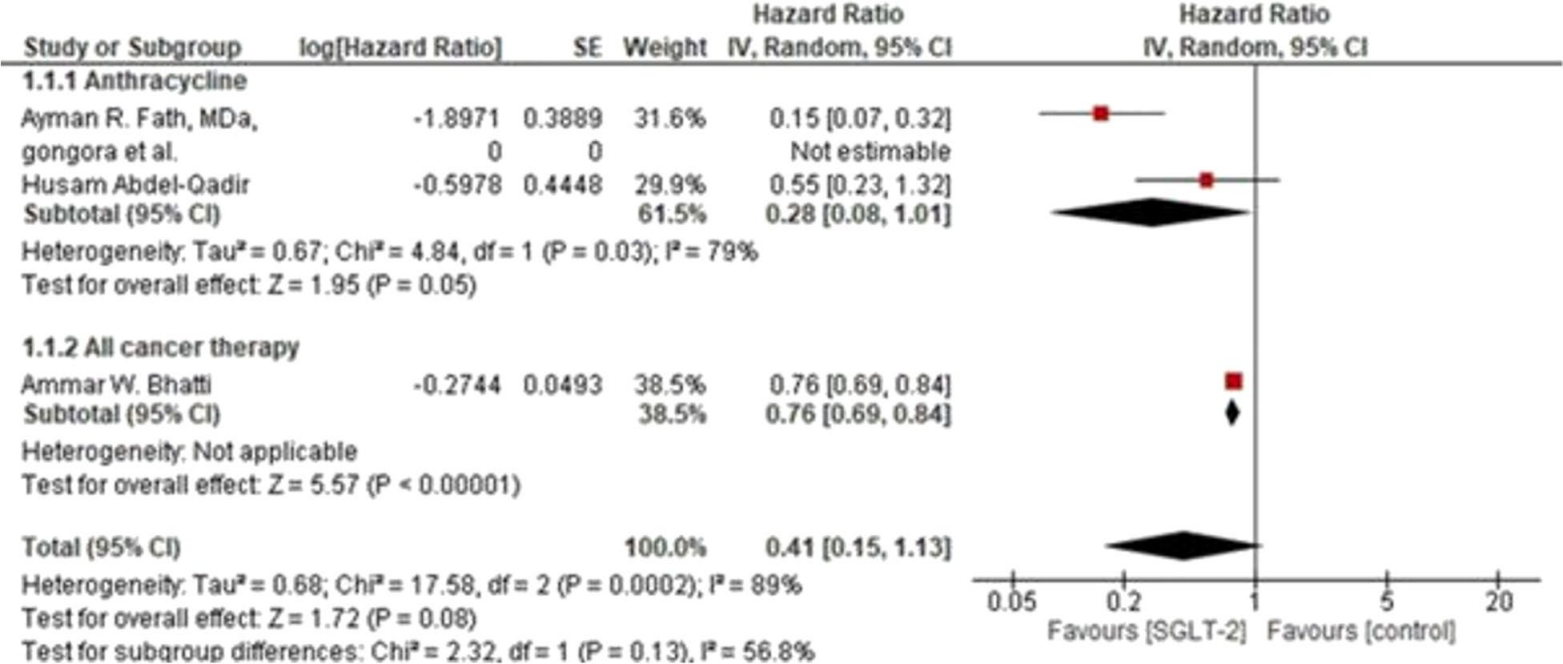
Forest plot of Hazard ratio of HF incidence

Forest plot showing meta-analysis of HRs and their corresponding 95% CIs for a subgroup analysis between anthracycline or all cancer therapy (including anthracycline) and the risk of developing cancer therapy–related HF incidence while on sodium glucose co-transporter 2 inhibitor (SGLT-2 inhibitors). We included three studies that compared the hazard ratio of HF incidence between SGLT-2 inhibitors and control (non-SGLT-2 inhibitors). Each subgroup demonstrated a potential advantage of SGLT-2 inhibitors with a reduced risk of heart failure incidence, either assessed for anthracycline or all cancer therapies.The difference in the number of trials and participants between the two subgroups indicates that the analysis was underpowered to detect meaningful differences. In the pooled analysis, there was no significant difference in the HF incidence between the groups (HR 0.41, 95% CI: 0.15 to 1.13; *p* = 0.08). There was also significant heterogeneity observed (*P* = 0.0002; I² = 89%; τ² = 0.68). Sensitivity analyses resolved heterogeneity (OR 0.55; 95% CI, 0.30 to 1.02; *P* = 0.06; studies = 2; I² = 0%).(Fig. 6: Forest plot of Hazard ratio of HF incidence).

### Hazard ratio of HF exacerbation

Forest plot showing meta-analysis of HRs and their corresponding 95% CIs for a subgroup analysis between anthracycline or all cancer therapy (including anthracycline) and the risk of developing cancer therapy–related HF exacerbation while on sodium glucose co-transporter 2 inhibitor (SGLT-2 inhibitors). We included three studies that compared the hazard ratio of HF exacerbation between SGLT-2 inhibitors and control (non-SGLT-2 inhibitors). Each subgroup demonstrated a potential advantage of SGLT-2 inhibitors with a reduced risk of heart failure exacerbation, either assessed to anthracycline or all cancer therapy.

**Fig. 7:**
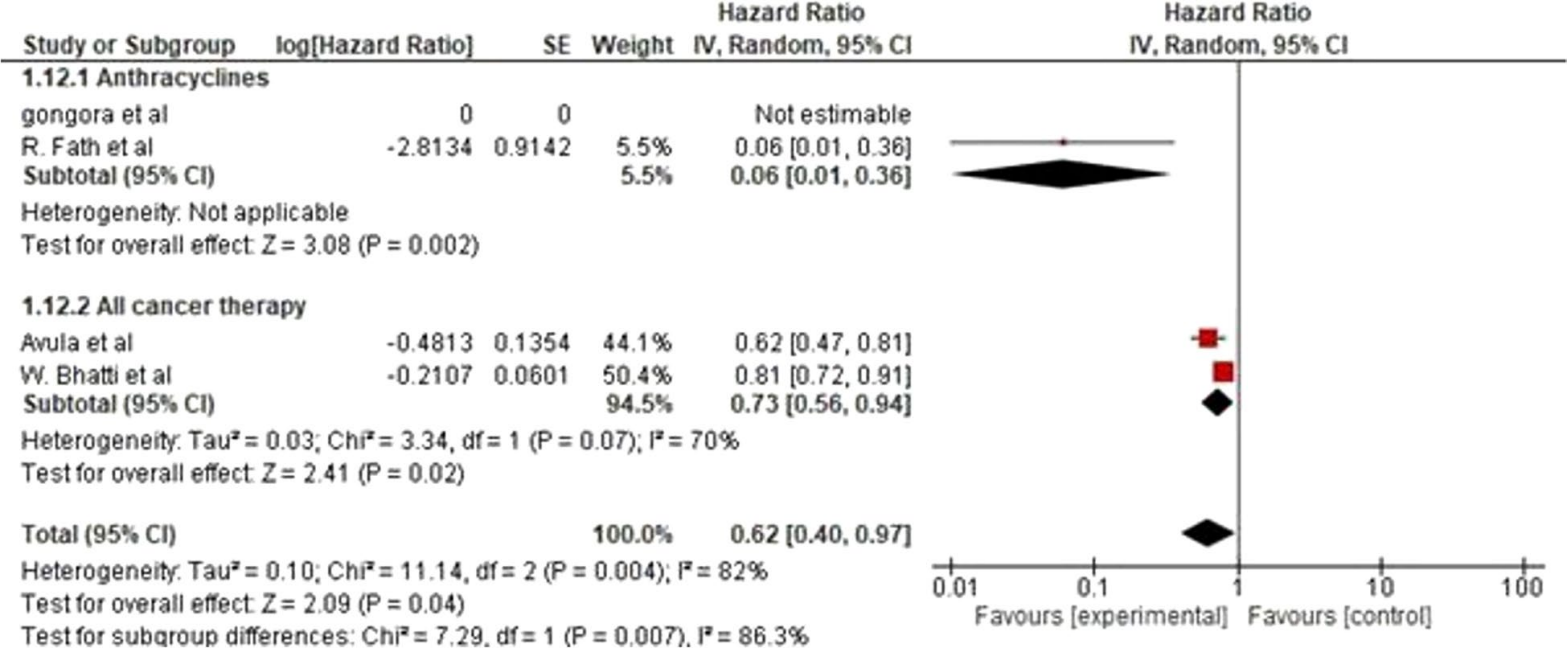
Forest plot of Hazard ratio of HF exacerbation

A subgroup analysis was performed to test whether being assessed to anthracycline or all cancer therapy “including anthracycline” modifies the cardioprotective effect of SGLT-2 inhibitors in comparison to non-SGLT-2 inhibitors regarding risk of HF exacerbation. However, the difference in the number of trials and participants between the two subgroups means that the analysis was underpowered to detect meaningful differences. In the pooled analysis, the three studies found that the SGLT-2 inhibitors group was associated with a significantly 38% lower risk of developing HF exacerbation compared to the non-SGLT-2 inhibitors group in patients exposed to all cancer therapy and anthracycline alone, with a hazard ratio (HR) of 0.62 (95% CI: 0.40–0.97; *P* = 0.04). There was also significant heterogeneity observed (*P* = 0.004; I² = 82%; τ² = 0.10). (Fig. 7: Forest plot of Hazard ratio of HF exacerbation).

### Hazard ratio of all-cause hospitalizations

Forest plot showing meta-analysis of HRs and their corresponding 95% CIs for a subgroup analysis between anthracycline or all cancer therapy, including anthracycline, and the risk of developing cancer therapy–related hospitalization while on sodium glucose co-transporter 2 inhibitor (SGLT-2 inhibitors).

We included three studies that compared the hazard ratio of all-cause hospitalizations between SGLT-2 inhibitors and control (non-SGLT-2 inhibitors). The difference in the number of trials and participants between the two subgroups means that the analysis was unpowered to detect meaningful differences. The pooled analysis found that the all-cause hospitalization risk in patients exposed to all cancer therapy and anthracycline alone is lower in the SGLT-2 inhibitors group than in the non-SGLT-2 inhibitors group; however, the difference between the two groups was not statistically significant (HR: 0.86, 95% CI: 0.70–1.06; *P* = 0.16). There was also significant heterogeneity observed (*P* < 0.0001; I² = 87%; τ² = 0.03). (Supplementary Figure: Hazard Ratio of all-cause hospitalizations Forest plot)

### Hazard ratio of atrial fibrillation

We included two studies involving patients receiving all cancer therapy that compared the hazard of atrial fibrillation between SGLT-2 inhibitors and control (non-SGLT-2 inhibitors) groups. The pooled analysis under a random-effects model demonstrated that the SGLT-2 inhibitor group was associated with a statistically significant 30% reduction in the risk of atrial fibrillation (HR 0.70, 95% CI: 0.54 to 0.90; *p* = 0.006). No significant heterogeneity was observed (I² = 26%, *p* = 0.26). (Supplementary Figure: Hazard Ratio of Atrial Fibrillation Forest plot)

## Discussion

### Overview of findings

This systematic review and meta-analysis aim to evaluate the cardioprotective effect of oral antidiabetic drugs (SGLT-2 inhibitors, metformin, and GLP-1 receptor agonists) in diabetic cancer patients treated with chemotherapy, particularly those with anthracycline-induced cardiotoxicity. This analysis compares outcomes between diabetic cancer patients treated with chemotherapy and oral antidiabetic drugs versus diabetic cancer patients treated with chemotherapy without oral antidiabetic drugs. The analysis revealed that oral antidiabetic drugs, especially SGLT-2 inhibitors, are associated with significant therapeutic benefits, including reduced all-cause mortality, atrial fibrillation, HF exacerbation, HF incidence, arrhythmia, and all-cause hospitalization, especially regimens containing anthracyclines. While metformin and GLP-1 receptor agonists were included, limited study numbers precluded reliable subgroup conclusions for these agents.

This meta-analysis shows that the cardioprotective properties of diabetic drugs in oncological patients have a hierarchy of evidence as the strength of association of each outcome decreases inversely by the heterogeneity of the observed values. The strongest evidence was the use of sodium-glucose co-transporter-2 (SGLT-2 inhibitors) as a predictor of the atrial fibrillation outcome of hazard ratio, which showed a significant relative risk of 30% (HR 0.70, 95% CI 0.54–0.90; *p* = 0.006) with no measurable heterogeneity (I²=26). The odds ratio analysis of arrhythmia incidence specific to the subgroup of SGLT-2 inhibitors showed a well-established 65% odds ratio reduction (OR 0.35, 95% CI 0.20–0.60; *p* = 0.0002), and no heterogeneity (I² = 0%).

The pooled analyses demonstrated statistically significant benefits of large magnitudes but a significant degree of heterogeneity (I² = 75–81%) in the case of critical outcomes such as all-cause mortality, heart failure (HF) exacerbation, and HF incidence. Diabetic medication use was also related to a 50% decrease in all-cause mortality (OR 0.50, 95% CI 0.39736; *p* = 0.00001), a 42% decrease in HF exacerbation (OR 0.58, 95% CI 0.45736; *p* = 0.0001), and a 64% decrease in the incidence of new-onset HF (OR 0.36, 95% CI 0.28736; *p* = 0.0001). Sensitivity analyses were used to support these findings, and they showed that the omission of studies that were found to represent significant sources of bias and heterogeneity (Gongora et al. and Fath et al.) did not affect the statistical significance of such pooled estimates, further supporting the strength of the core cardioprotective signal.

Additional analysis in terms of prediction intervals gave an essential context of generalizability. In the case of mortality, the 95% prediction interval (0.25 to 1.34) showed that the actual effect in a new environment is almost entirely positive, however, with a somewhat varying magnitude. The uncertainty was greater concerning HF incidence (prediction interval 0.03–4.64), indicating that the average of such effects is highly protective, but the outcome in a new population may theoretically be both a deep decrease to a possible increase in risk and a clinical and methodological heterogeneity. Results such as all-cause hospitalization (OR 0.73) and atrial fibrillation (by odds ratio, OR = 0.61) have equally beneficial effects but were also equally characterized by huge heterogeneity (I²=100%), with heterogeneous effect sizes across the studies included.

On the other hand, some of the analyses produced non-significant or very inconsistent results, mainly because the heterogeneity is very high. The HF incidence hazard ratio was neither statistically significant (HR 0.41, 95% CI 0.15–1.13; *p* = 0.08) nor was it heterogeneous (I²=89%). Similarly, the all-cause hospitalization hazard ratio was not significant in risk reduction (HR: 0.86, 95% CI: 0.70–1.06; *P* = 0.16), and the heterogeneity was high (I²=87%). These results show that there is not enough evidence at present to make a reliable conclusion of a time-dependent, consistent protective effect of these endpoints. In conclusion, there is strong evidence in favor of SGLT-2 inhibitors in the prevention of arrhythmias and atrial fibrillation, moderate but less reliable evidence regarding mortality prevention and heart failure, and minimally conclusive evidence regarding hospitalization risk reduction with time in the presence of SGLT-2 inhibitors.

### Heterogeneity and interpretation of effect sizes

The fact that we found a high heterogeneity among studies included in our meta-analysis is an intrinsic factor influencing the way we are going to interpret our meta-analytic results. This inconsistency, indicated by statistically significant I² statistics and the p-value of the Q tests of most of our pooled results, was a natural result of the nature of the source data, which was observational. The heterogeneity probably stems from a combination of clinical and methodological factors, such as changes in the baseline demographics of patients (e.g., age, hypertension prevalence), the types and stages of cancer, specific anthracycline agents and cumulative doses, the types of SGLT-2 inhibitors and GLP-1 receptor agonists, differences in follow-up, and geographical differences in clinical practice. Instead of disregarding this heterogeneity, we used a multi-faceted statistical method to quantify, explore, and contextualize it and enhance the quality of the validity and clinical usefulness of our findings.

First of all, to account for this variability, the analysis was done using random-effects models [23–25]. Then, subgroup analysis is used to try to solve heterogeneity. The stratified analysis by the type of diabetic medication (SGLT-2 inhibitors, metformin, and GLP-1 receptor agonists) allowed us to conclude that the strongest and most stable signal of cardioprotection was produced by the SGLT-2 inhibitors. In outcomes such as all-cause mortality, atrial fibrillation, and heart failure exacerbation, SGLT-2 inhibitor subgroups not only showed statistically significant advantages but also, in certain cases, showed a varying degree of effect compared to the overall pool. This stratification demonstrated that a large part of the total heterogeneity was explained by the combination of pharmacologically different drug classes. In the same way, when results of hazard ratios are concerned, we conducted subgroup analyses by chemotherapy exposure (anthracycline-only vs. all cancer therapy containing anthracyclines). Although these analyses were generally underpowered to find statistically significant differences between subgroups, they gave important suggestions on whether the SGLT-2 inhibitor treatment effect was consistent across different clinical settings, indicating that the benefit of SGLT-2 inhibitors was not limited to pure anthracycline-based regimens but could be extended to more general chemotherapeutic exposures.

In order to measure quantitatively the strength of our pooled estimates and directly deal with the impact of methodological outliers, we did sensitivity analyses. This was an important step in our study of heterogeneity. In particular, we successively filtered out the studies listed as the principal sources of bias and variability, i.e., Gongora et al. (its risk of bias due to unadjusted confounders is too high) and Fath et al. (it is a major contributor to statistical heterogeneity). This removal of the studies resulted in a large reduction of the I² statistic on the most important outcomes, including all-cause mortality and heart failure exacerbation, usually curing the large heterogeneity, and the pooled effect remained statistically significant. (Supplementary Figures: sensitivity analysis)

Last, and most importantly, to apply our findings to clinical practice, we estimated 95% prediction intervals of the major findings. When the confidence interval (CI) is used to measure the accuracy around the average treatment effect, the prediction interval (PI) is used to measure the interval within which the true treatment effect of a similar study that will occur in the future is likely to lie. It is a more realistic approach where there is a presence of heterogeneity. In the case of our major outcome of all-cause mortality, the pooled odds ratio was 0.50 (0.39 to 0.63), and the average benefit is significant. The prediction interval, however, was much broader, as it was between 0.24 and 1.06, which is a 95% prediction interval. This interval provides a subtle tale: whereas the balance of evidence indicates a mortality effect (except that most of it lies below 1.0), it recognizes the fact that in one or more future clinical situations or patient groups, the effect may be inconsequential (almost all 1.0) or even harmful (even more than 1.0, but the lower extreme). Equally, in the case of HF exacerbation (PI: 0.25 to 1.34) and HF incidence (PI: 0.03 to 4.64), the large prediction intervals literally measure the extensive uncertainty in the generalizability of the effect size. Making these intervals report shifts the discussion out of a single summary statistic and necessitates a more careful and advanced interpretation, which stresses that the actual cardioprotective effect is not a point value but a distribution of potential effects. Most importantly, this uncertainty also translated into the HR analyses, which give an evaluation of risk over time. The mortality pooled HR was 0.52, 95% CI: 0.39–0.70; *P* < 0.0001, although its 95% prediction range of 0.17 to 1.59 indicates that a future study had a possibility of discovering a risk reduction as much as 83% or as small as a 59% higher risk. Likewise, the prediction interval of the HR of HF exacerbation (HR 0.62, 95% CI: 0.40–0.97) was large (implied by high heterogeneity), and their HR of HF incidence was so heterogeneous that it did not impact significantly. The significant breadth of these intervals is a quantitative measure in itself of the remaining heterogeneity, which remains even after our other tests. It brings on board the uncertainty presented by clinical and methodological differences in all studies. Thus, though our sensitivity analyses endorse the strength of the positive direction of the effect, the prediction intervals offer an important qualification on the variable strength of this effect and that the actual cardioprotective benefit is not a number but a distribution. Such frank calculation of uncertainty, based on our overall heterogeneity analysis, reinforces the methodological rigor of our review and underscores the need to do more standardized, prospective studies that could bring this range of effects estimated to a smaller range. (Supplementary figures: Prediction interval plots)

In summary, the heterogeneity in our analysis is not a mere statistical footnote but a central feature of the evidence base. By the concerted use of subgroup, sensitivity, and prediction interval analysis, we have not simply recognized this heterogeneity but have been busy deconstructing it. This procedure proves that the cardioprotective advantages discovered are strong and resilient, remaining despite the variations in analytical processes and assumptions. The similarity of the direction of effect between subgroups and sensitivity analyses, in combination with the largely positive range of the interval of prediction, is a strong, although probabilistic, case in favor of the possible role of these medications. In the end, these approaches never eradicate heterogeneity but offer a clear setup for studying the implications of the latter, thus producing both statistically and clinically meaningful conclusions.

### Consistency with previous literature

Our findings are strongly supported by three recent, high-quality meta-analyses. Spadafora et al. (2025) provide strong confirmation of our main results, showing similar reductions in all-cause mortality (RR: 0.38) and heart failure outcomes (RR: 0.52). Importantly, there is no heterogeneity (I² = 0%) that indicates that the cardioprotective effect of SGLT2 inhibitors becomes clearer as more evidence accumulates. They also found a significant reduction in arrhythmias (RR: 0.40), further reinforcing the broad cardiovascular benefits of this drug class [26]. Tabowei et al. (2024), who focused on patients receiving anthracycline-based chemotherapy, reported mortality benefits (RR: 0.55) that closely match our results. Their non-significant finding for heart failure incidence (RR = 0.67, 95% CI: 0.40–1.41) mirrors the variability seen in our analysis, suggesting that small sample sizes and limited statistical power may explain the inconsistent results across studies [27]. Novo et al. (2025) also reported comparable effect sizes for key outcomes, including all-cause mortality (RR: 0.47), heart failure hospitalization (RR: 0.44) further strengthening the overall pattern of evidence [28]. Taken together, the consistent results across these independent analyses provide strong and converging evidence that SGLT2 inhibitors exert meaningful cardioprotective effects in this high-risk population.

### Cardioprotective mechanisms and clinical impact of SGLT-2 inhibitors

To better understand the observed cardioprotective benefits of SGLT-2 inhibitors in Diabetic cancer patients, it is essential to explore the underlying mechanisms through which these agents exert their effects. Preclinical studies suggest that SGLT2 inhibitors exert a broad spectrum of cardioprotective effects that may prevent cardiotoxicity. Studies have demonstrated that SGLT2 inhibitors limit mitochondrial dysfunction by restoring calcium homeostasis, reducing reactive oxygen species, and lipid peroxidation (LPO) [29–31]. 

Moreover, SGLT-2 inhibitors improve Activation of AMPK-mTOR, which regulates autophagy. SGLT-2 inhibitor directly reduced expression of pro-inflammatory cytokines (IL-6, IL-8, and IL-1), NLRP3, MyD88, and NF-kB, which decrease cardiac inflammation and fibrosis [30, 32, 33].

Furthermore, SGLT-2 inhibitors enhance mitochondrial function, dynamics, and metabolic efficiency [34, 35]. These findings suggest the role of SGLT-2 inhibitors in reversing the anthracycline-induced cardiotoxicity.

### Study limitation

This meta-analysis has several important limitations that should be considered when interpreting the results. First, only retrospective cohort studies were included, which may introduce potential biases such as exposure bias and selection bias, thereby limiting causal inference. Second, the generalizability of the findings may be restricted due to heterogeneity in study designs, sample sizes, patient characteristics (including demographic and regional differences), and treatment protocols across the included studies. Additionally, variations were observed in the type of SGLT-2 inhibitor used, the timing of administration relative to anthracycline therapy, and the duration of follow-up, all of which could influence the reported outcomes. We addressed between-study heterogeneity by conducting subgroup analyses and applying prediction intervals to better account for variability across studies. Furthermore, the control groups typically consisted of patients on other glucose-lowering therapies, meaning the observed cardioprotective effect is a comparison against other active drugs rather than a placebo or no treatment, which could influence the magnitude of the estimated benefit.

## Conclusion

This meta-analysis suggests that SGLT-2 inhibitors may be associated with a reduced risk of all-cause mortality, atrial fibrillation or flutter, heart failure (HF) incidence and exacerbation, arrhythmia, and all-cause hospitalization in patients receiving chemotherapy-associated cardiotoxic agents. Metformin use was linked to a lower risk of hear t failure incidence and all-cause mortality, while GLP-1 receptor agonists showed a potential reduction in the risk of all-cause mortality, HF exacerbation, all-cause hospitalization, atrial fibrillation, and arrhythmia. To validate and strengthen these findings, further large-scale, high-quality randomized controlled trials (RCTs) are needed.

## Supplementary Information

Supplementary Material 1.

## Data Availability

All the data are available when the editor requests.

## Abbreviations

SGLT2 inhibitors: Sodium-Glucose Transporter-2 Inhibitors
GLP-1 receptor agonists: Glucagon-Like Peptide-1
DM: Diabetes Mellitus
ANTs: Anthracyclines
AIC: Anthracycline-Induced Cardiotoxicity
CTRCD: Cancer Therapy-Related Cardiac Dysfunction
CCBs: Calcium Channel Blockers
HF: Heart Failure
AF: Atrial Fibrillation
LVEF: Left Ventricular Ejection Fraction
AMI: Acute Myocardial Infarction
RCT: Randomized Controlled Trial
PRISMA: Preferred Reporting Items for Systematic Reviews and Meta-Analyses
PICO: Patient/Population, Intervention, Comparison, and Outcome (Framework)
MeSH: Medical Subject Headings
OR: Odds Ratio
HR: Hazard Ratio
PI: Prediction interval
CI: Confidence Interval
ROBINS-I: Risk Of Bias In Non-randomized Studies of Interventions
GRADE: Grading of Recommendations, Assessment, Development and Evaluation
ROB: Risk of Bias
ATT-PSM: Average Treatment effect on the Treated - Propensity Score Matching
PSM: Propensity Score Matching
