# Supplementary material for: Cardioprotective effect of diabetic medication on cancer patients undergoing proven cardiotoxic chemotherapy: a systematic review and meta-analysis
Source: Cardiooncology. 2026 Jan 15;12:14. doi: 10.1186/s40959-025-00424-4 (PMC12853835; doi:10.1186/s40959-025-00424-4)
Supplement: Supplementary file 1 — Supplementary Material 1. [file 40959_2025_424_MOESM1_ESM.zip › 40959_2025_424_MOESM8_ESM.docx]

| **Study ID** | **Outcomes Definition according to the paper** | **ESC Cardio-oncology outcomes Definition** |
| --- | --- | --- |
| Abdel-Qadir 2023 | HF incidence: new diagnosis of HF in- or out-of-hospital. Hospital admission for HF1. OR two claims for HF recorded in the National Ambulatory Care Reporting System and/or the Ontario Health Insurance Plan (OHIP) physician claims database12. These two claims must occur within 365 days of each other  HF exacerbation: hospitalization with a most responsible (primary) diagnosis of HF  Arrhythmia incidence: Not reported | HF incidence: new or worsening myocardial dysfunction, often classified by left ventricular ejection fraction (LVEF) changes. Typically, it relates to a reduction in LVEF below 50% or significant deterioration in cardiac biomarkers or myocardial strain even when asymptomatic.  HF exacerbation: sudden worsening or acute decompensation of heart failure symptoms requiring urgent medical attention.  Arrhythmia incidence: new onset or worsening arrhythmias during or after cancer therapy, with certain cancer drugs. |
| Bhatti et al. 2024 | HF incidence: Incident CTRCD was defined using International Classification of Diseases-10th Revision (ICD-10) codes for new onset cardiomyopathy or HF  HF exacerbation: not reported but defined using International Classification of Diseases-10th Revision (ICD-10) codes  Arrhythmia incidence: not reported but defined using International Classification of Diseases-10th Revision (ICD-10) codes |  |
| Avula et al. 2024 | HF incidence: not reported  HF exacerbation: HF exacerbations were defined by ICD-10 codes OR the need for intravenous loop diuretics  Arrhythmia incidence: incidence Atrial fibrillation and flutter but not reported but defined using International Classification of Diseases-10th Revision (ICD-10) codes |  |
| Fath et al. 2024 | HF incidence: New-onset HF was specifically defined as a diagnosis of new-onset systolic, diastolic, or combined HF  HF exacerbation: Not reported  Arrhythmia incidence: New-onset arrhythmia was explicitly defined as atrial fibrillation or atrial flutter (AF/AFL) and/or ventricular arrhythmia |  |
| Gongora et al. 2022 | HF incidence: was specifically defined as a decline in left ventricular ejection fraction (EF) to <53%  HF exacerbation: Identified with two identities that are not specific - Heart Failure Exacerbation Outpatient and HF Admissions.  Arrhythmia incidence: clinically significant arrhythmias |  |
| Hwang et al. 2023 | HF incidence: The study's cohort excluded patients with a current or previous diagnosis of heart failure (HF) or cardiomyopathy. The outcome tracked for severe heart failure events was HF hospitalization.  HF exacerbation: not reported  Arrhythmia incidence: not reported |  |
| Osataphan et al. 2023 | HF incidence: Not reported but the most similar outcome was Chemotherapy-Related Cardiac Dysfunction (CTRCD) and was defined as an Left Ventricular Ejection Fraction (LVEF) reduction of more than 10% to the value below 53%.  HF exacerbation: Not reported  Arrhythmia incidence: Not reported |  |
| Vignarajah et al. 2025 | HF incidence: Not reported but the most similar outcome was Chemotherapy-Related Cardiac Dysfunction (CTRCD) and was defined as an Left Ventricular Ejection Fraction (LVEF) reduction of more than 10% to the value below 53%  HF exacerbation: HF exacerbations were identified by ICD-10 codes or the need for intravenous loop diuretics  Arrhythmia incidence: no reported outcome but the study stated AF and VF incidence outcomes |  |
| Onoue et al. (2023) | HF incidence: new onset symptomatic HF occurring within 1 year of the initiation of anthracyclines  HF exacerbation: Not Reported  Arrhythmia incidence: Not reported |  |
| Chiang et al (2023) | HF incidence : hospitalization for incident heart failure (HF)  HF exacerbation: Not reported  Arrhythmia incidence: Not reported |  |
| Scalia et al (2025) | HF incidence :acute heart failure (including either new diagnosis or episode of acute on chronic heart failure)  HF exacerbation: Not reported  Arrhythmia: new onset atrial fibrillation or flutter |  |
