# Supplementary figures and images for: Cardioprotective effect of diabetic medication on cancer patients undergoing proven cardiotoxic chemotherapy: a systematic review and meta-analysis
Source: Cardiooncology. 2026 Jan 15;12:14. doi: 10.1186/s40959-025-00424-4 (PMC12853835; doi:10.1186/s40959-025-00424-4)

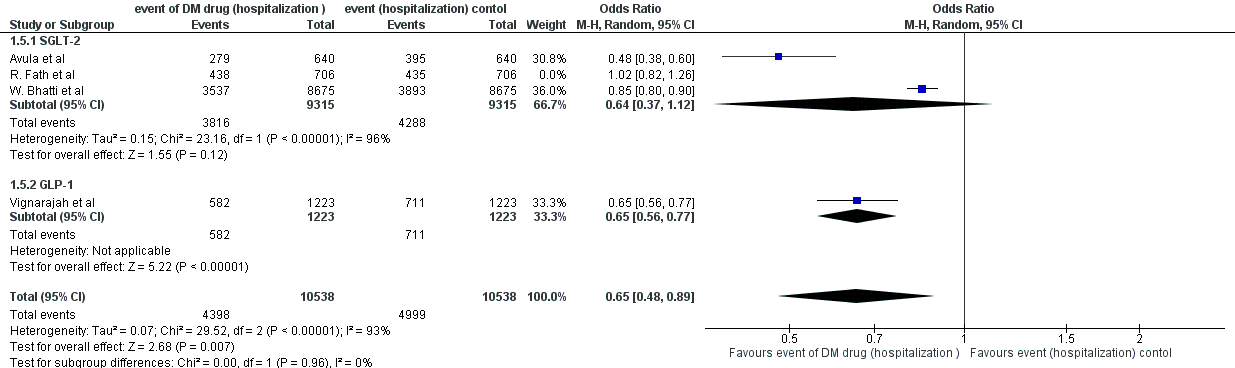

Supplement: Supplementary file 1 — Supplementary Material 1. [file 40959_2025_424_MOESM1_ESM.zip › 40959_2025_424_MOESM3_ESM.png]

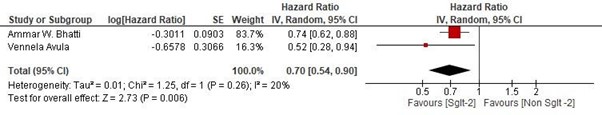

Supplement: Supplementary file 1 — Supplementary Material 1. [file 40959_2025_424_MOESM1_ESM.zip › 40959_2025_424_MOESM1_ESM.jpg]

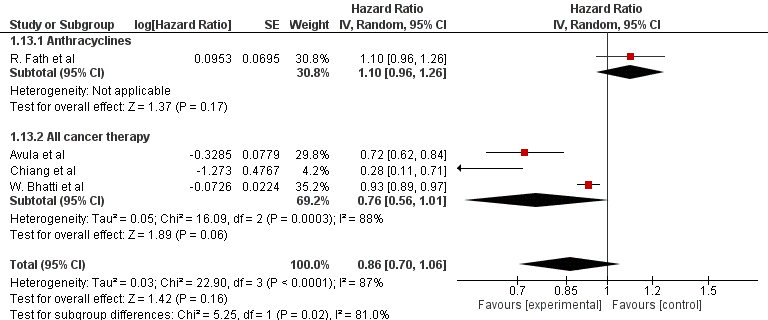

Supplement: Supplementary file 1 — Supplementary Material 1. [file 40959_2025_424_MOESM1_ESM.zip › 40959_2025_424_MOESM2_ESM.png]

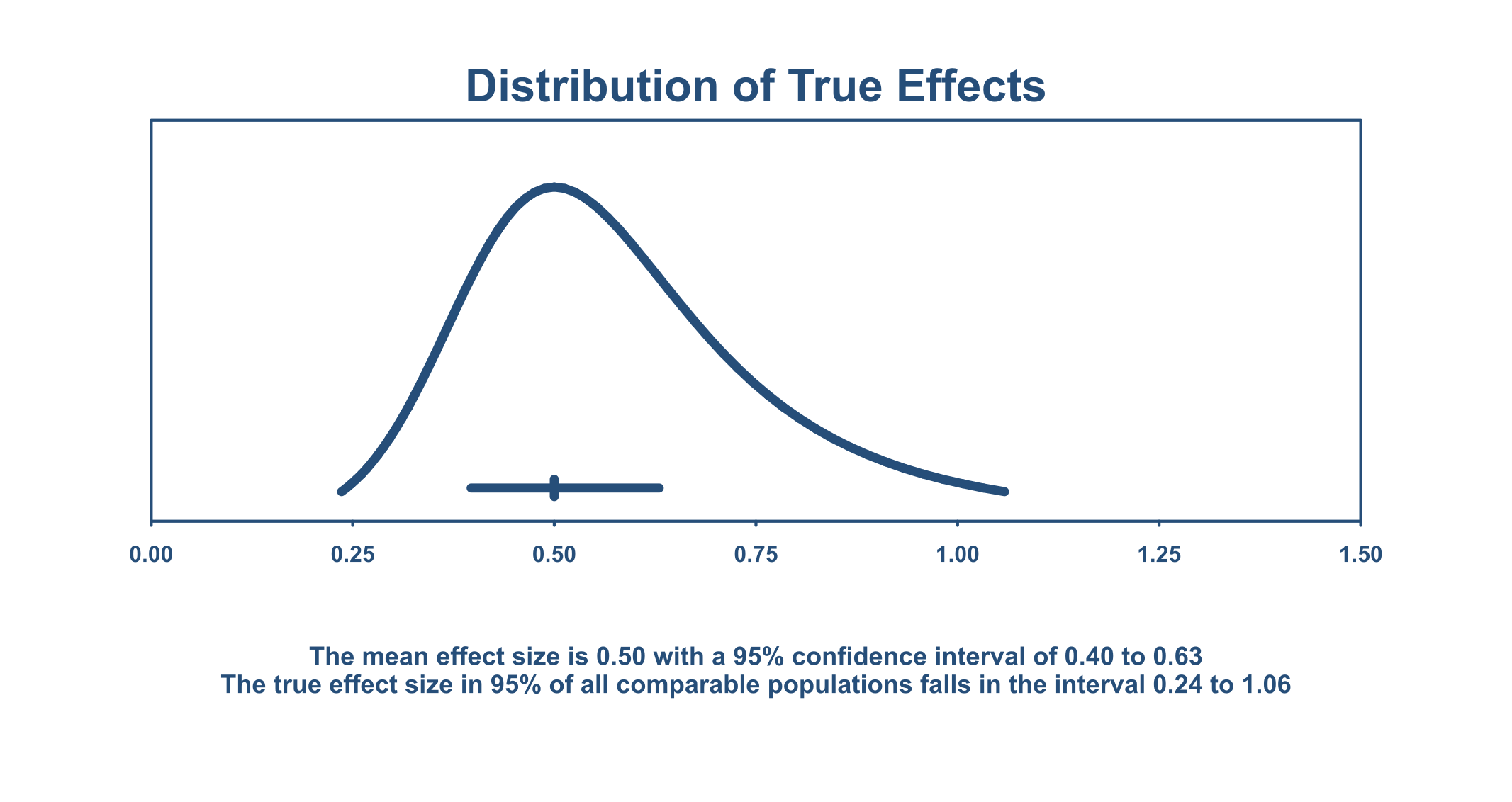

Supplement: Supplementary file 1 — Supplementary Material 1. [file 40959_2025_424_MOESM1_ESM.zip › 40959_2025_424_MOESM11_ESM.png]

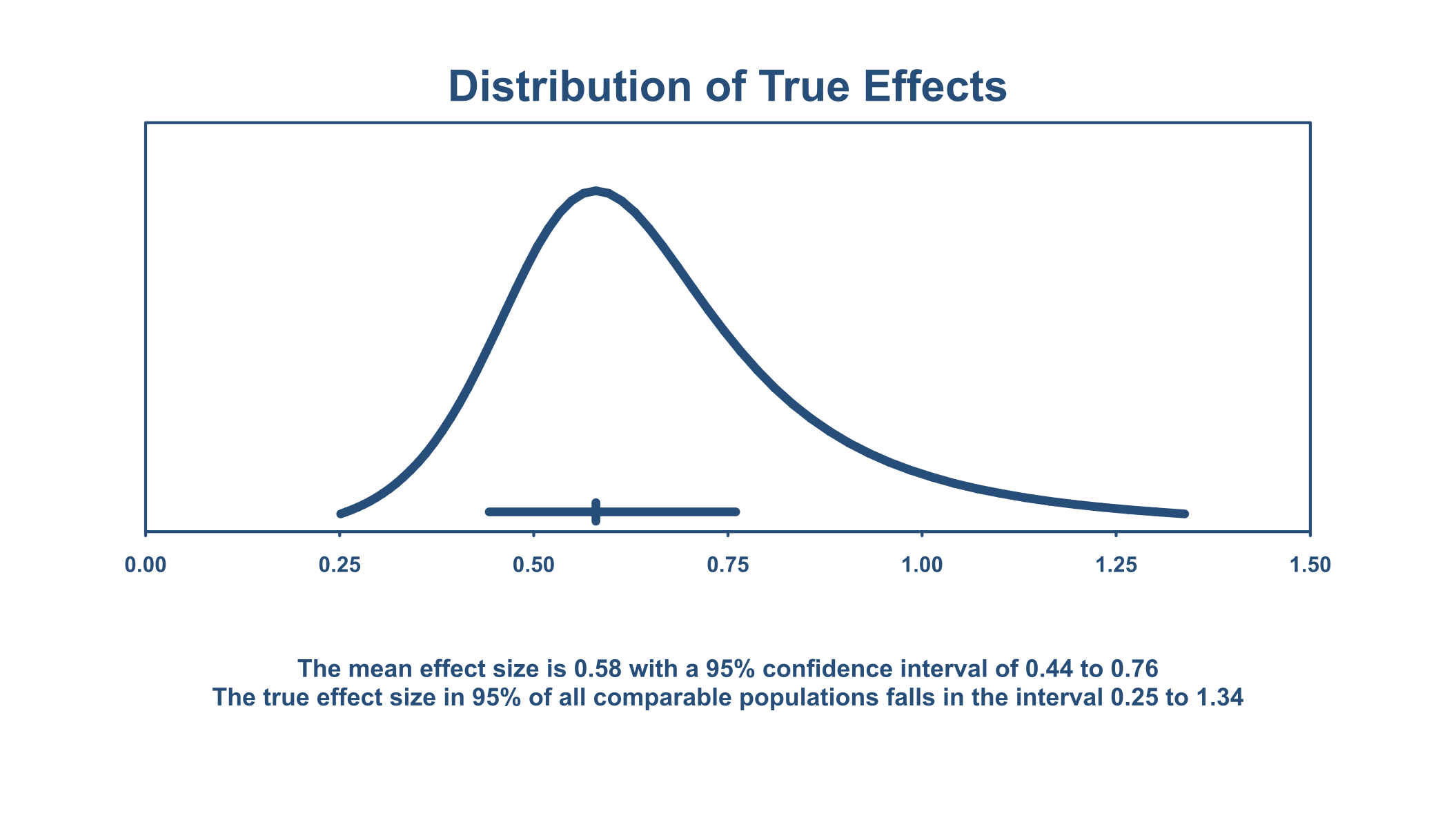

Supplement: Supplementary file 1 — Supplementary Material 1. [file 40959_2025_424_MOESM1_ESM.zip › 40959_2025_424_MOESM12_ESM.png]

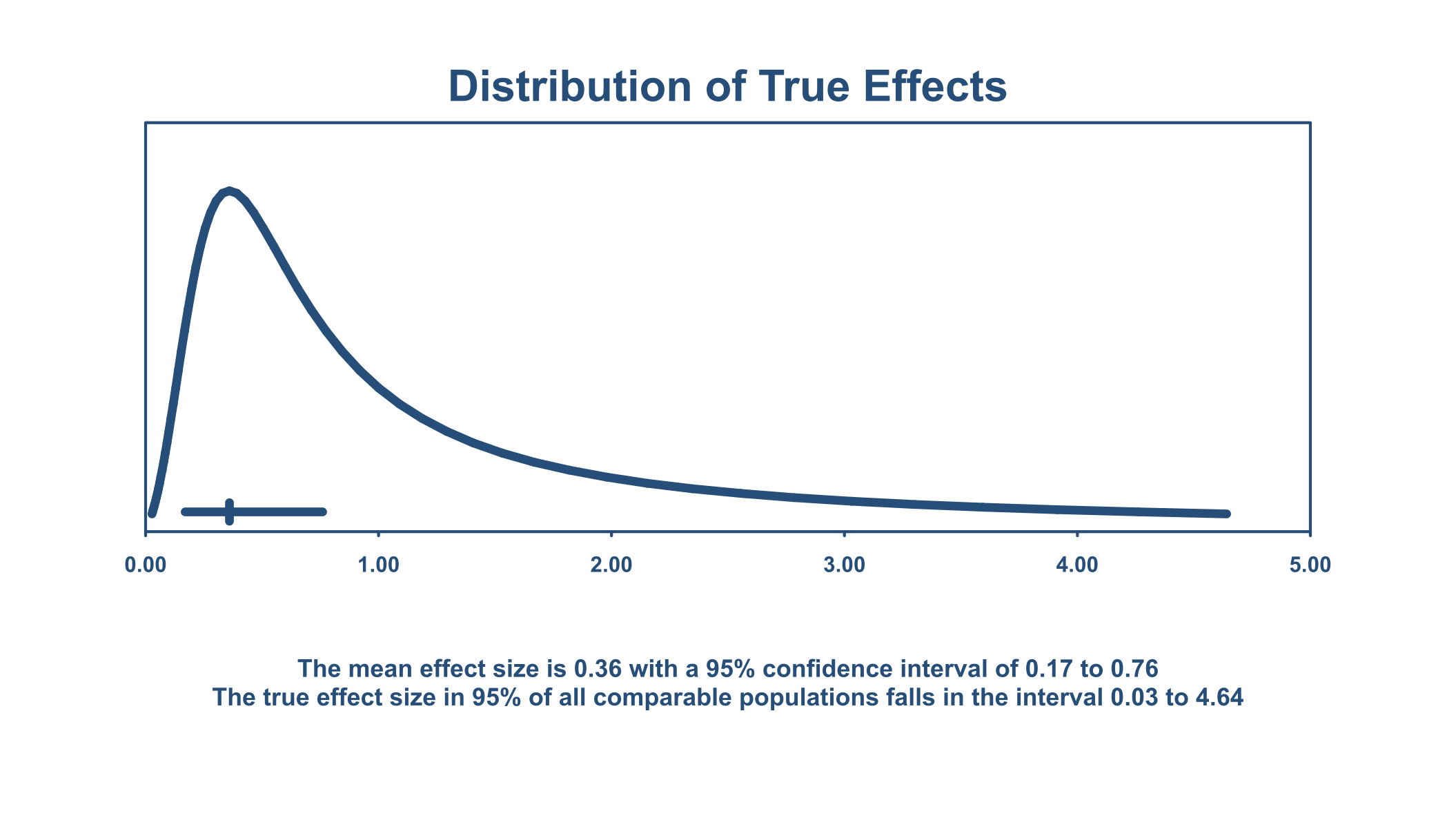

Supplement: Supplementary file 1 — Supplementary Material 1. [file 40959_2025_424_MOESM1_ESM.zip › 40959_2025_424_MOESM10_ESM.png]

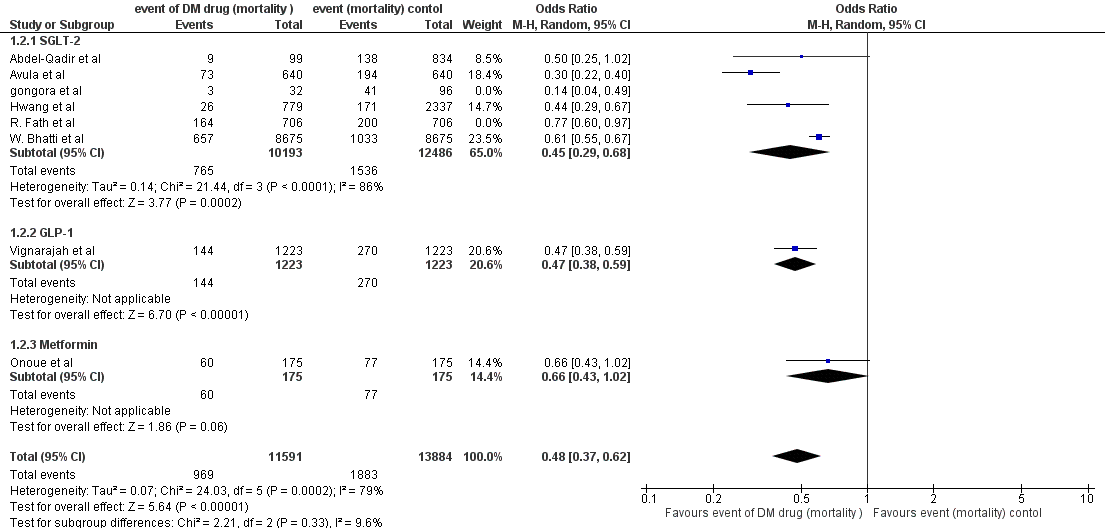

Supplement: Supplementary file 1 — Supplementary Material 1. [file 40959_2025_424_MOESM1_ESM.zip › 40959_2025_424_MOESM9_ESM.png]

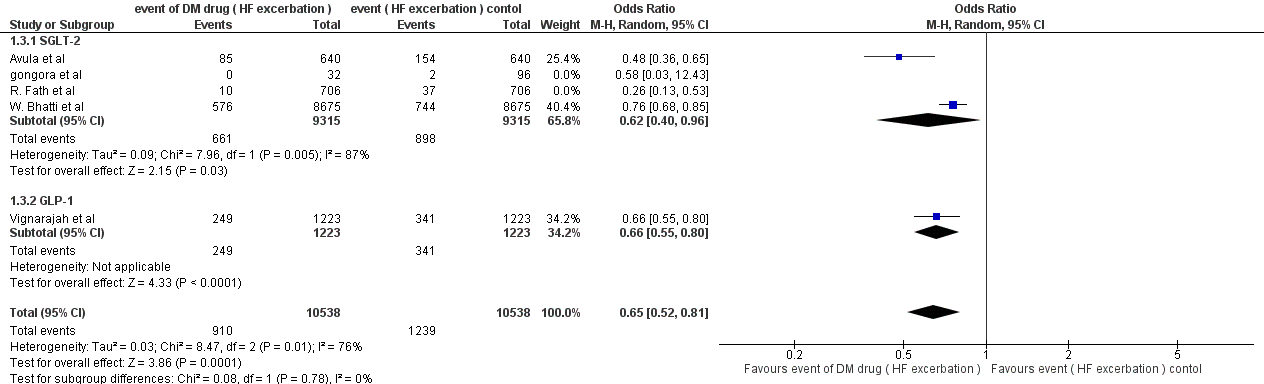

Supplement: Supplementary file 1 — Supplementary Material 1. [file 40959_2025_424_MOESM1_ESM.zip › 40959_2025_424_MOESM7_ESM.png]

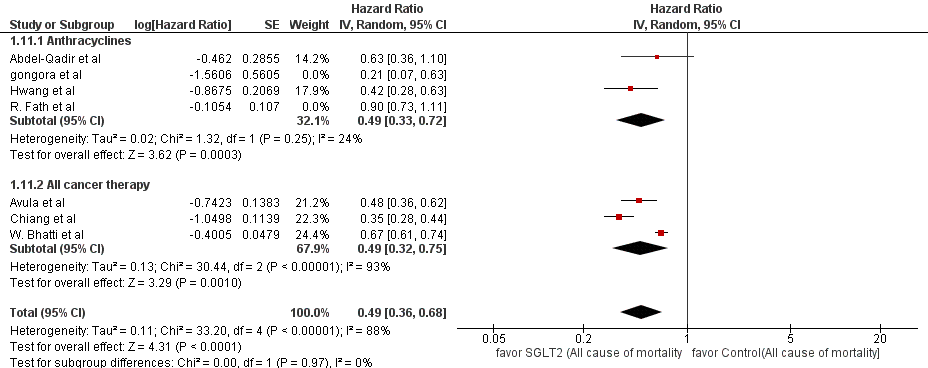

Supplement: Supplementary file 1 — Supplementary Material 1. [file 40959_2025_424_MOESM1_ESM.zip › 40959_2025_424_MOESM6_ESM.png]

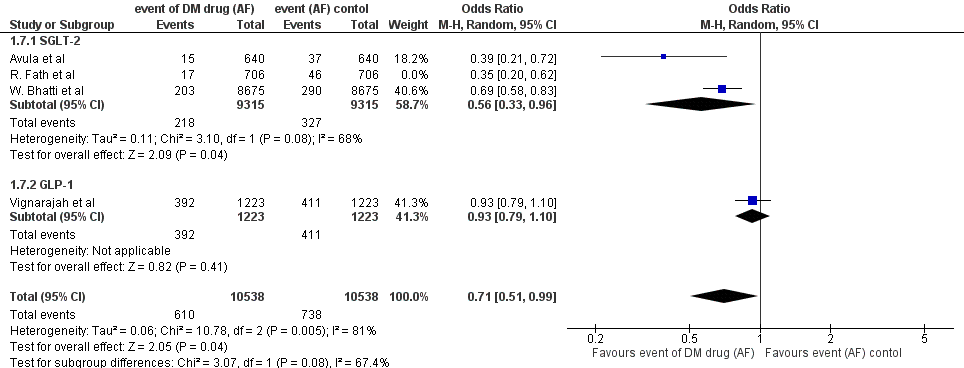

Supplement: Supplementary file 1 — Supplementary Material 1. [file 40959_2025_424_MOESM1_ESM.zip › 40959_2025_424_MOESM5_ESM.png]

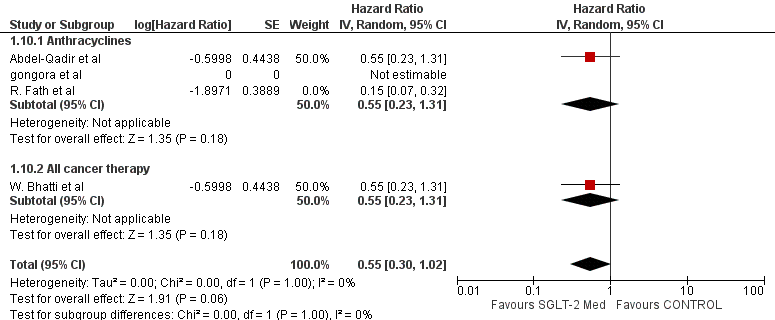

Supplement: Supplementary file 1 — Supplementary Material 1. [file 40959_2025_424_MOESM1_ESM.zip › 40959_2025_424_MOESM4_ESM.png]
